# Structure-Based Design and Pharmacophore-Based Virtual Screening of Combinatorial Library of Triclosan Analogues Active against Enoyl-Acyl Carrier Protein Reductase of *Plasmodium falciparum* with Favourable ADME Profiles

**DOI:** 10.3390/ijms24086916

**Published:** 2023-04-07

**Authors:** Cecile Bieri, Akori Esmel, Melalie Keita, Luc Calvin Owono Owono, Brice Dali, Eugene Megnassan, Stanislav Miertus, Vladimir Frecer

**Affiliations:** 1 Laboratoire de Physique Fondamentale et Appliquée (LPFA), University of Abobo Adjamé (Now Nangui Abrogoua), Abidjan 02, Côte d’Ivoire; 2 Department of Physics, Ecole Normale Supérieure, University of Yaoundé I, P.O. Box 47, Yaoundé 1, Cameroon; 3 International Centre for Applied Research and Sustainable Technology, SK-84104 Bratislava, Slovakia; 4 International Centre for Theoretical Physics, Strada Costiera 11, I-34151 Trieste, Italy; 5 Laboratoire de Cristallographie—Physique Moléculaire, Université De Cocody, Abidjan 22, Côte d’Ivoire; 6 Laboratoire de Chimie Organique Structurale et Théorique, Université De Cocody, Abidjan 22, Côte d’Ivoire; 7 Department of Biotechnologies, Faculty of Natural Sciences, University of SS. Cyril and Methodius, SK-91701 Trnava, Slovakia; 8 Department of Physical Chemistry of Drugs, Faculty of Pharmacy, Comenius University Bratislava, SK-83232 Bratislava, Slovakia

**Keywords:** malaria, *Pf*ENR, triclosan, molecular modelling, QSAR, pharmacophore, virtual combinatorial library, in silico screening, molecular dynamics, ADME-related properties

## Abstract

Cost-effective therapy of neglected and tropical diseases such as malaria requires everlasting drug discovery efforts due to the rapidly emerging drug resistance of the plasmodium parasite. We have carried out computational design of new inhibitors of the enoyl-acyl carrier protein reductase (ENR) of *Plasmodium falciparum* (*Pf*ENR) using computer-aided combinatorial and pharmacophore-based molecular design. The Molecular Mechanics Poisson–Boltzmann Surface Area (MM-PBSA) complexation QSAR model was developed for triclosan-based inhibitors (TCL) and a significant correlation was established between the calculated relative Gibbs free energies of complex formation $\left(∆∆G_{com}\right)$ between *Pf*ENR and TCL and the observed inhibitory potencies of the enzyme $\left({IC}_{50}^{exp}\right)$ for a training set of 20 known TCL analogues. Validation of the predictive power of the MM-PBSA QSAR model was carried out with the generation of 3D QSAR pharmacophore (PH4). We obtained a reasonable correlation between the relative Gibbs free energy of complex formation ${∆∆G}_{com}$ and ${IC}_{50}^{exp}$ values, which explained approximately 95% of the *Pf*ENR inhibition data: ${{pIC}_{50}^{exp}=-0.0544\times {∆∆G}_{com}+6.9336,R}^{2}=0.95$. A similar agreement was established for the PH4 pharmacophore model of the *Pf*ENR inhibition (${pIC}_{50}^{exp}=0.9754\times {pIC}_{50}^{pre}+0.1596$, $R^{2}=0.98$). Analysis of enzyme–inhibitor binding site interactions suggested suitable building blocks to be used in a virtual combinatorial library of 33,480 TCL analogues. Structural information derived from the complexation model and the PH4 pharmacophore guided us through in silico screening of the virtual combinatorial library of TCL analogues to finally identify potential new TCL inhibitors effective at low nanomolar concentrations. Virtual screening of the library by *Pf*ENR-PH4 led to a predicted ${IC}_{50}^{pre}$ value for the best inhibitor candidate as low as 1.9 nM. Finally, the stability of *Pf*ENR-TCLx complexes and the flexibility of the active conformation of the inhibitor for selected top-ranking TCL analogues were checked with the help of molecular dynamics. This computational study resulted in a set of proposed new potent inhibitors with predicted antimalarial effects and favourable pharmacokinetic profiles that act on a novel pharmacological target, *Pf*ENR.

## 1. Introduction

Malaria is a major disease in tropical areas, where about 40% of the world’s population lives. This disease is responsible for about 0.6 million deaths per year, 98% of which are in Africa. According to the WHO, children under 5 years of age account for 77% of all malaria deaths [1]. *Plasmodium falciparum* (*Pf*) infection is the most virulent form of malaria, accounting for most malaria cases worldwide [1]. The significant spread of resistance to traditional antimalarial drugs underscores the need to develop new potent and orally bioavailable antimalarial agents, which are also active against existing drug-resistant *P. falciparum* strains [2].

Recently reported cases of resistance of *Pf* to artemisinin and combined therapies (ACT) in Rwanda, Uganda, and Eritrea [3,4] as well as in Burkina Faso and Mali [5] worsen the picture and make the discovery of new antimalarials for Africa more urgent. Finally, almost 400 years after the discovery of quinine (Cinchona bark) originating in South America and 40 years after the introduction of artemisinin (*Artemisia annua*) from Asia, eyes are fixed on Africa and finding a novel molecule from the rich pharmacopoeia of this continent, which will help overcome the problem of the resistance of *Pf* to artemisinin and its derivatives. Currently, numerous validated pharmacological targets for *Pf* inhibition have been identified at different stages of the complex parasite life cycle, many of them not yet involved in the parasite’s resistance [6,7]. In fact, the treatment of parasite infection in the erythrocytic stage is preferred for most approved antimalarials, in contrast to the liver stage, where administered medical preparations serve primarily for prophylactic purposes [8,9].

The synthesis of fatty acids is an essential process for all living organisms. Fatty acids play a critical role in the supply of metabolic precursors of biological membranes and represent an important form of metabolic energy production. Attention is being given to inhibition of the type II fatty acid biosynthesis pathway (FAS-II), which is different from the related FAS-I pathway present in mammalian cells. FAS-II of *Pf* appears to hold significant promise in the discovery of novel drugs, which are expected to be less toxic to humans due to the difference between these two distinct pathways [10,11,12,13]. Enoyl-acyl carrier protein reductase (ENR) is a crucial enzyme in the type II fatty acid synthesis pathway of many pathogens, including *Plasmodium falciparum*, the etiologic agent of malaria. The final reaction in the FAS-II pathway of *Pf* is catalysed by enoyl-acyl carrier protein reductase (*Pf*ENR), which mediates the NADH-dependent reduction of the *trans*-2-enoyl-acyl carrier protein [14,15].

**Figure 1:**
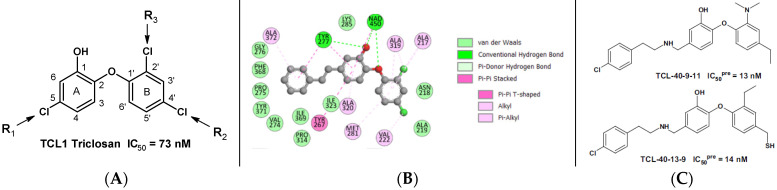
Chemical structure of **TCL1** triclosan. (**A**) scaffold atom numbering and the positions of the R-group are indicated. (**B**) Structure of **TCL11** (${IC}_{50}=$ 76 nM, Table 1) and 2D interaction diagram with residues from the active site of *Pf*ENR (PDB entry 2OOS) [21]. Atom colouring: C—grey, O—red, Cl—green. (**C**) triclosan analogues that were previously predicted to be potent inhibitors of *Pf*ENR [6,32].

Triclosan (**TCL1**, Figure 1), a common antibacterial agent incorporated into a variety of consumer products, is an uncompetitive inhibitor of purified *Pf*ENR, which has demonstrated inhibitory potency against *P. falciparum* parasites cultured in vitro $\left({IC}_{50}^{exp}=73nM\right)$ [16,17,18]. The **TCL1** derivatives (Figure 1) are non-toxic to mammalian cells [17,19] which do not contain ENR homologues. The human exposure routes, metabolism, and toxicity of triclosan have recently been reviewed with a conclusion that adverse biological effects of triclosan and its degradation products need to be investigated [20]. Freundlich previously reported that **TCL1** analogues inhibit both *Pf*ENR enzymes and *Mycobacterium tuberculosis* ENR enzymes [21,22,23,24,25]. *Pf*ENR is a well-known validated pharmacological target of antimalarials [26]. The existence of X-ray crystal structures of **TCL1** and its analogues plus the NAD^+^ cofactor bound to *Pf*ENR facilitate the possibility of computer-assisted drug design of antimalarial agents, which target lipid synthesis in the plasmodium parasite [18,21]. In the bound conformation, the aromatic rings of **TCL1** are orthogonal to each other, the phenol ring mimicking the enolate intermediate of the reaction catalysed by the ENR. Simultaneously the **TCL1** is face to face π-stacked with NAD+, T-shaped π-stacked with the catalytic Tyr 277, and forms hydrogen bonds to the cofactor and the tyrosine, respectively [21]. Replacements of chlorine atoms at positions 5, 2′ and 4′ on the **TCL1** diarylether scaffold by larger groups resulted in energetically favourable interactions with both the enzyme and the co-factor [22,23,27,28]. Synthesis of 4′-derivatives of **TCL1** caused minor gains in the inhibitory potencies towards *Pf*ENR [27], while the analogues substituted in the 2′ position reached only the micromolar range of the ${IC}_{50}s$ [23]. Sullivan et al. [28] described the synthesis and inhibitory activity tests of 5-alkyl-substituted **TCL1** derivatives toward purified *Pf*ENR. Chibber et al. [22] studied TCL analogues with mainly hydrophilic 5-substituents in carbon 5, which were less potent than **TCL1**. Freundlich et al. [21] reported on the synthesis and activity tests of **TCL1** analogues containing hydrophobic substituents at position 5 intended to better ‘root’ the inhibitor at the active site of *Pf*ENR. Their results demonstrated the possibility of improving the in vitro antimalarial potency of **TCL1** by replacing the suboptimal 5-chloro group with larger hydrophobic moieties. The **TCL1** scaffold has inspired numerous initiatives to design potent *Pf*ENR inhibitors in a search for new antimalarials [29,30,31]. In our previous work, a QSAR model of TCL binding to *PfENR* was built using the LUDI scoring function to allow the in silico screening of a virtual combinatorial library of 120 **TCL1** analogues with favourable predicted ADME profiles [32]. The best designed TCLs reached predicted ${IC}_{50}^{pre}$ slightly over 10 nM (Figure 1). So far, few 3D-QSAR pharmacophore (PH4) studies have been reported for the inhibition of *PfENR* by TCL. The PH4 pharmacophore was used for **TCL1** in complex with *Pf*ENR and based on receptor coordinates recovered from the X-ray structure 3F4B (Protein Data Bank) [33]. Unfortunately, this PH4 model is spatially limited, since 5′-Cl is not large enough to fill the surrounding hydrophobic pocket of the enzyme active site. The search for a reliable PH4 model taking into account the reported SAR of *Pf*ENR inhibition for the known TCL inhibitors [9,14,15,16,18] seems tricky, as illustrated in the following comparative analysis of the role of 5′-substituents: **TCL1** ($R_{1},R_{2},R_{3}\equiv$ Cl, Cl, Cl; 73 nM), **TCL10** ($R_{1},R_{2},R_{3}\equiv$ CH_2_–Ph, Cl, Cl; 71 nM), **TCL11** ($R_{1},R_{2},R_{3}\equiv$ (CH_2_)_2_–Ph, Cl, Cl; 76 nM), and **TCL15** ($R_{1},R_{2},R_{3}\equiv$*p*F–Ph, Cl, Cl; 38 nM). Extension of the –(CH_2_)_n_– linker to fill the hydrophobic pocket next to C-5 is not advantageous, while the *p*-fluorophenyl of **TCL15** increases the potency twice, in contrast to linkers. Therefore, the design of new more potent TCLs against *Pf*ENR requires careful application of the 3D-QSAR PH4 model, attention to the active conformation, and also exploration of the pockets of the active site for the positions C2′ and C4′ of the TCL scaffold.

In this work, we further develop predicted inhibitory potencies of TCLs against *Pf*ENR by extended combinatorial design and a virtual screening approach:

- We built and validated a Hansch-type QSAR model to correlate the relative Gibbs free energy (rGFE) of TCL binding to *Pf*ENR with the observed inhibitory potencies $\left({IC}_{50}^{exp}\right)$ (identification of the bound conformation of TCL inhibitors).
- The robustness of the built model is confirmed by a 3D-QSAR pharmacophore (PH4) model based on the bound conformations of the training set of inhibitors (generation of the PH4 model).
- In addition, we built a virtual library of 33,480 analogues of TCLs and screened them with the help of PH4 to identify hits for inhibition of *Pf*ENR (virtual screening).
- Finally, the ADME profile of the best designed analogues was predicted and compared with those of current antimalarial drugs and compounds undergoing clinical trials (postprocessing step 1).
- Each of the hits was cross-checked for predicted inhibitory potency by computed rGFE of the formation of the *Pf*ENR-TCLx complex, which led to the identification of a handful of prospective novel TCLs that exceeded the potency of known TCLs against *Pf*ENR (postprocessing step 2).
- The top 5 TCL hits and **TCL11** underwent molecular dynamics simulations to explore the stability of the *Pf*ENR-TCLx complexes (postprocessing step 3).

## 2. Results

### 2.1. Training and Validation Sets of TCL Inhibitors

The training set (20 molecules) and validation set (4 molecules) of triclosan analogues that inhibit *Pf*ENR of the malaria parasite (Table 1) used in this study were selected from the literature [21,23,25,27]. The inhibitory potencies of these derivatives cover a sufficiently broad range of activities to allow a reliable QSAR model to be built $\left(38nM\leq {IC}_{50}^{exp}\leq 10^{5}nM\right)$.

### 2.2. QSAR Model of PfENR Inhibition

#### 2.2.1. Single-Descriptor QSAR Model of *Pf*ENR Inhibition by TCLs

For each of the 20 TS and 4 VS molecules (Table 1), an enzyme-inhibitor complex *Pf*ENR-TCLx was constructed by in situ modifications of the refined template crystal structure of the complex *Pf*ENR-**TCL11** (PDB entry code 2OOS [21]) as described in Section 4. Furthermore, the relative Gibbs free energy (rGFE) of the complex formation (${∆∆G}_{com}$) and its components was calculated for each of the 24 geometry-optimised *Pf*ENR-TCLx complexes for the triclosan derivatives (Table 2; see footnote of Table 2 for definitions of the quantities). A QSAR model that correlates the computed rGFE with the experimental inhibitory potencies of TCLs (${pIC}_{50}^{exp}=-\log_{10}{IC}_{50}^{exp}$ [17]) explained 95% of the variation in the observed activities. The resulting linear regression equation is shown in Table 3, Equation (2). Relatively high values of the regression coefficient $R^{2}$, leave-one-out cross-validated regression coefficient $R_{xv}^{2}$ and Fischer F-test of the correlation suggest a strong relationship between the 3D model of inhibitor binding and the observed inhibitory potencies of TCL [21]. Therefore, structural information derived from the 3D models of *Pf*ENR-TCLx complexes can be expected to lead to a reliable prediction of the inhibitory potencies of *Pf*ENR for new TCL analogues based on QSAR Equation (2) (Table 3). To gain a better understanding of the binding affinity of TCLs to *Pf*ENR, we have analysed the enthalpy of complexation in gas phase ${∆∆H}_{MM}$ by correlating it with the ${pIC}_{50}^{exp}$. The validity of the resulting linear correlation (for statistical data from the regression, see Table 3, Equation (1)) allowed the assessment of the contribution of inhibitor-enzyme interactions (${∆∆H}_{MM}$) when the solvent effect and loss of inhibitor entropy upon binding to the enzyme were neglected. This correlation explained about 94% of the ${pIC}_{50}^{exp}$ data variation and underlined the role of interatomic interactions in determining the binding affinity of the ligands. These statistically significant correlations allowed us to reveal the bound conformations of the TCLs at the *Pf*ENR binding site and allowed the definition of the PH4 pharmacophore.

**Figure 2:**
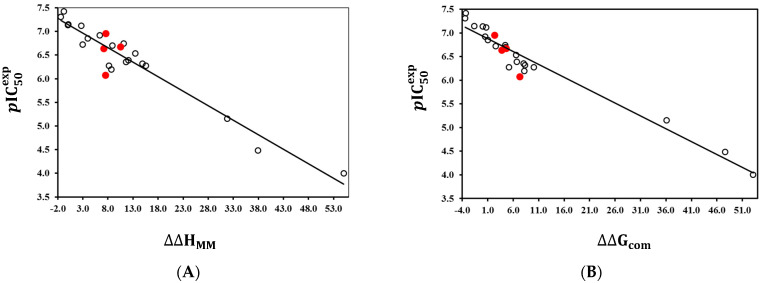
(**A**) graph of the correlation Equation (1) (Table 3) between ${pIC}_{50}^{exp}$ and relative enthalpic contribution ${∆∆H}_{MM}$ [kcal·mol^−1^] to the rGFE. (**B**) plot of the rGFE of the *Pf*ENR-TCLs complex formation ${∆∆G}_{com}$ [kcal·mol^−1^] (Equation (2), Table 3) of the training and validation sets [21]. The data points from the validation set are shown in red.

The statistical data confirmed the validity of the correlation Equations (1) and (2) shown in Figure 2. The ratio ${pIC}_{50}^{pre}/{pIC}_{50}^{exp}≅1$ calculated for the validation set TCL21–24 documents the substantial predictive power of the QSAR model (the ${pIC}_{50}^{pre}$ values were estimated from computed ${∆∆G}_{com}$ using the correlation Equation (2), Table 3). Thus, the regression Equation (2) and computed ${∆∆G}_{com}$ (Table 2) can be used to predict inhibitory potencies against *Pf*ENR for new TCL analogues, provided that they share the same binding mode as the training set of triclosan derivatives **TCL1–20**.

#### 2.2.2. Binding Mode of TCLs to *Pf*ENR

**Figure 3:**
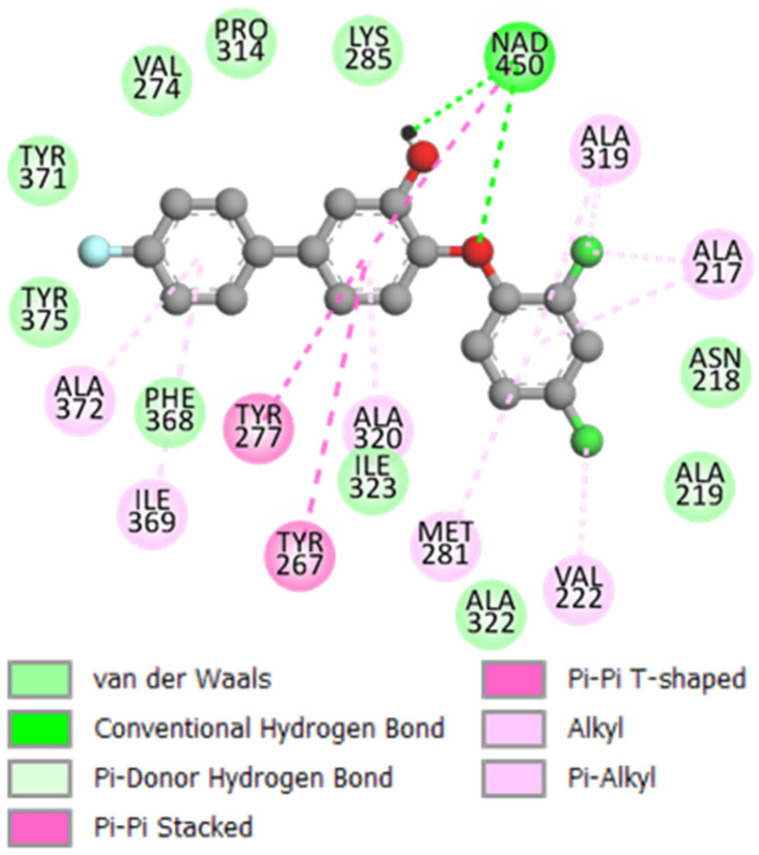
2D scheme of interactions of the most potent *Pf*ENR inhibitor **TCL15** (${IC}_{50}^{exp}=$ 38 nM, [9,17]) with the residues of the reductase active site.

**Figure 4:**
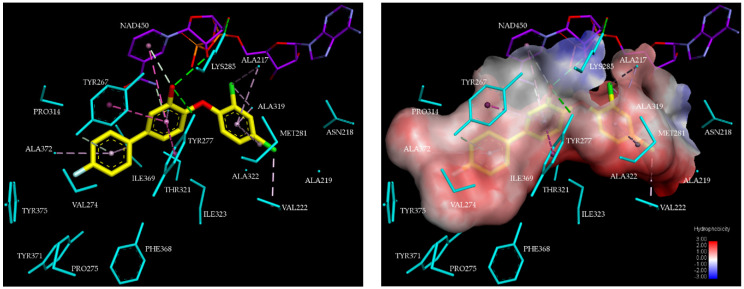
(**Left**) 3D structure of the active site with the most potent inhibitor **TCL15** (${IC}_{50}^{exp}=$ 38 nM, [21]). The carbon atoms are coloured yellow; the residue side chains and carbon atoms of the NAD cofactor are coloured cyan and purple, respectively. Interaction colour code: hydrogen bonds (green), hydrophobic contacts of alkyl groups (pink), π -π interactions (dark pink). (**Right**) Molecular surface of the active site of *Pf*ENR. Surface colouring legend: red, hydrophobic; blue, hydrophilic; white, intermediate.

The binding mode of TCLs derived from the crystal structure [21] and the QSAR model are illustrated in Figure 3 and Figure 4. The interactions of the TCL inhibitor at the active site of *Pf*ENR present in the reference X-ray structure of the **TCL11** complex (Figure 3, [21]), namely the 𝜋-𝜋 stacking between the phenol moiety and the nicotinamide ring of ${NAD}^{+}$, are conserved. Furthermore, hydrogen bonds (HB) involving the hydroxyl group of TCL and Tyr277, as well as the ribose ring of ${NAD}^{+}$ (Figure 3 and Figure 4), are also preserved.

One of the main contributions of TCL derivatives to the enzyme-inhibitor interaction in *Pf*ENR involves the hydrophobic pocket formed by the residues Tyr267, Val274, Tyr277, Tyr375, Phe368, Ala269, Ala320, Ala372, and Ile369 [21,32]. As we can see in Figure 4, for inhibitor **TCL15** the hydrophobic $R_{1}$ substituent Ph −*p*F reaches the hydrophobic pocket and contributes significantly to the stabilising interactions at the active site of *Pf*ENR. The $R_{2}$ group of TCL analogues is surrounded by active site residues Asn218, Val222, Met281, and substrate binding loop residues Ala319 and Ile323.

Further enrichment of enzyme-inhibitor interactions by new substitutions in **TCL1** analogues with the same mode of *Pf*ENR binding will lead to improved potencies of the new structurally similar compounds, provided that the predictive power of correlation Equation (2) also extends beyond the range of activities of the training set. The predictive ability of our QSAR model relying on the computed ${∆∆G}_{com}$ was also confirmed by the PH4 pharmacophore model of the *Pf*ENR inhibitory activity.

### 2.3. 3D-QSAR Pharmacophore Model of PfENR Inhibition

**Figure 5:**
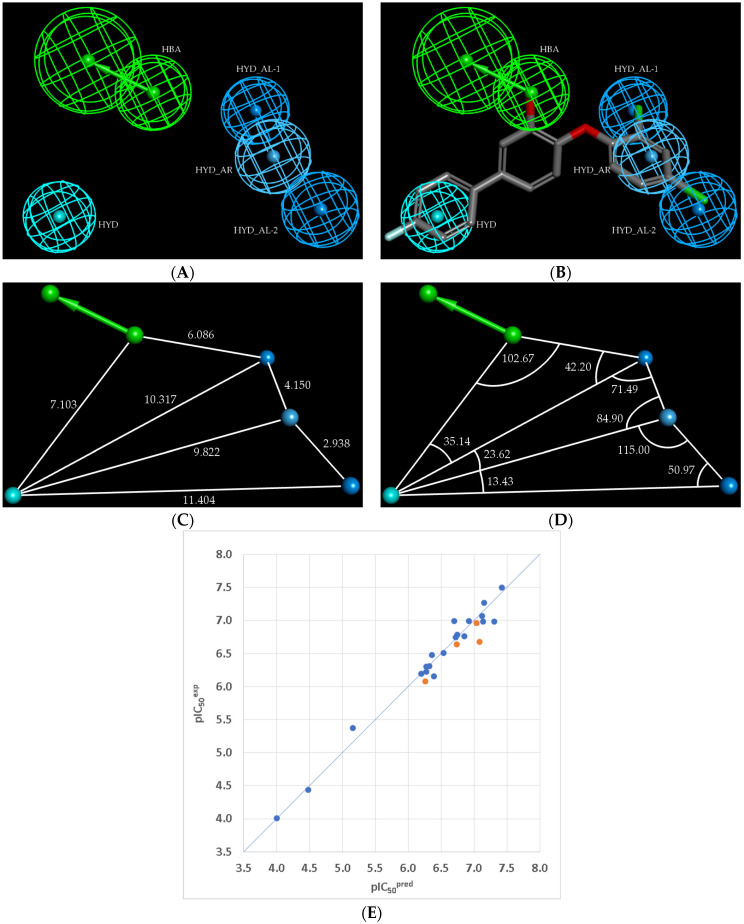
Features of the best PH4 model (Hypo1) of *Pf*ENR inhibitors generated by the 3D-QSAR pharmacophore module: (**A**) Coordinates of the centres, (**B**) mapping of Hypo1 with **TCL15** (the most potent TCL molecule of the training set), (**C**) Distances in Å between the centres of the pharmacophoric features, (**D**) Angles (deg) between the centres. Colour code of features: blue—hydrophobic aliphatic; green—hydrogen bond acceptor; cyan—hydrophobic aromatic. (**E**) Plot of the linear correlation of experimental vs. predicted inhibitory activity (blue circles correspond to the training set and orange to the validation set).

The virtual screening process is operated with a ‘chemical space navigator’ (Pharmacophore, PH4) built from the bound conformations of the most active of 20 TCLs recovered from the *Pf*ENR-**TCL1–20** complexes that served to develop the QSAR model. Unlike direct pharmacophore generation from small free (not bound) molecules, the HypoGen algorithm in the 3D-QSAR pharmacophore protocol of Discovery Studio (DS) [34] generates a PH4 in order to estimate the biological activity (${IC}_{50}$) of the 20 training set compounds. Their ${IC}_{50}^{exp}$ values span 3.42 orders of magnitude (from 38 to 10^5^ nM), which is very close to the recommended 3.5 for reliable PH4 generation. To generate PH4, the constructive step of the algorithm selects compounds with ${IC}_{50}^{exp}\leq 1.25\times 38\;nM$ in order to build the starting PH4 features; only **TCL15** (${IC}_{50}^{exp}=$ 38 nM) is retained for this purpose. The 3.5 orders of magnitude criterion also served to identify the TCL compounds to be considered inactive in the subtractive step of the PH4 generation (${IC}_{50}^{exp}>38\times 10^{3.5}nM=120167\;nM$). Therefore, none of the other compounds from **TCL1–20** were subtracted, which means that all the PH4 features generated from **TCL15** were conserved for the processing of the hypotheses. Finally, during the last PH4 generation step, namely the optimisation phase, the score of the PH4 hypotheses is improved. According to a detailed description in the literature [35] as well as in Section 4, four main features were identified: hydrophobic (HYD) complementary to enzyme lipid pockets, hydrogen bond donor (HBD), and acceptor (HBA) binding to active site residues by acceptor and donor hydrogen bonds, respectively. Virtually screened molecules were mapped to these four pharmacophore features. The output of the HypoGen process is the top 10 PH4 hypotheses ranked according to their statistical data as presented in Table 4. Among them, the hypothesis cost is one of the most pertinent: the metrics defines the hypothesis total cost as a linear combination of three components, namely the weight, the error, and the configuration cost (see Section 4). It spans a range from 409.3 (Hypo1) to 1231.8 (Hypo10), all in a monotonic increasing order. Since the total cost is 803.5 (almost twice that of Hypo1), Hypo1 is the closest to the fixed cost and is very far from the other nine models. For the entire set of 10 hypotheses, two extreme additional costs are computed: the fixed cost (lower limit) of 15.2 and the null cost (upper bound) of 5423.2; the difference Δ = 5408.1 between them is the first criterion of the analysis. The larger the value of Δ (Δ > 70), the more meaningful are the top hypothesis models with a statistical significance greater than 90%. The second indicator is the difference between the null cost and the total cost of each hypothesis which is higher than 4190, indicating that all the 10 hypothesis models are very far from the null cost. The third indicator is the configuration cost of 9.55, far below 17, which is the value it should not exceed [36]. The next descriptor is the root-mean-square deviation (RMSD) of each hypothesis. The RMSD value of Hypo1 is equal to 6.6, the lowest. Hypo2 reached a RMSD of 9.5. Finally, the correlation coefficient (R^2^) of the training set reached a value of 0.96 for Hypo1 and 0.93 for the following Hypo2. All generated hypotheses display HYD, HBA, and HBD features. Finally, the first hypothesis (Hypo1) was retained for the in silico screening of the virtual library of TCL analogues. The additional statistical parameters of the regression for ${pIC}_{50}^{exp}$ vs. ${pIC}_{50}^{pre}$ estimated from Hypo1 for the training set are: ${pIC}_{50}^{exp}=0.9754\times {pIC}_{50}^{pre}+0.1596$ (n=20, $R^{2}=0.98$, $R_{xv}^{2}=0.96$, F−test=713.56, σ=0.145, α>98%). The correlation is plotted in Figure 5 and is very close to a slope (1.02) of one and an intercept of 0.13 near the origin. The parameters reflect the guidance of the Organisation of Economic Cooperation and Development (OECD) about QSAR [37]. An additional verification is the mapping of the most potent inhibitor **TCL15** to the Hypo1 features as shown in Figure 5. In the same figure, the detailed geometry and position of the Hypo1 PH4 features of *Pf*ENR inhibition are presented. PH4 was externally evaluated by a validation set of 4 derivatives, **TCL21–24**, that belong to the same interval of inhibitory potencies as the compounds of the training set.

For each of the 10 hypotheses, a confidence level of 98% is retained. The number of scrambled runs required is obtained using the following equation $S=\left[1-\left(1+X\right)/Y\right]\times 100$ where the number of hypotheses (X) the total cost of which is below that of Hypothesis 1 (Hypo1), is equal to 0; the total number of HypoGen runs (initial + random runs) should be equal to 1 + 49. Replacement of these values in the equation results in $S=\left[1-\left(1+0\right)/\left(1+49\right)\right]\times 100=98\%$.

The validation of the statistical significance of each hypothesis model is carried out using Fisher’s randomisation method. The process consists of the randomisation of the observed biological ${IC}_{50}^{exp}$ for the 20 training set compounds. It checks whether the PH4 model is not the result of an incidental correlation between ${IC}_{50}^{exp}$ and the conformations of TCL compounds (CatScramble programme). As indicated above, for the confidence level of S = 98%, 49 sets are generated for 10 pharmacophore hypotheses from spreadsheets built by random relationships between the ${IC}_{50}^{exp}$ and 3D structures of the **TCL1–20**. From the lowest cost among the 49 hypotheses for each of the 10 PH4 (Hypo1–Hypo10) listed in Table 4 (closest random), none shows a cost lower than the 409.3 of Hypo1. Randomisation confirms that Hypo1 is not produced by chance and is a true correlation supported by a statistically robust pharmacophore model for inhibition of *Pf*ENR by TCL with a confidence level of 98%. It shows predictive power as the QSAR complexation model on the ligand affinity descriptor ${∆∆G}_{com}$.

Next, we have carried out the computational design and selection of new TCL analogues with predicted elevated inhibition potencies against the malaria parasite *Pf*ENR, based mainly on the presence of the hydrophobic feature (HYD, Figure 5) in the best PH4 model at the substitution position of the $R_{1}$ group (see Table 1) and two hydrophobic aliphatic features located at the $R_{2}$ and $R_{3}$ positions. (HYD_AL-2 and HYD_AL-1, Figure 1 and Figure 5).

### 2.4. Virtual Library Generation and In Silico Screening of PfENR Inhibitors

In silico screening of a virtual combinatorial library can lead to hit identification, as was shown in our previous work on inhibitor design [32].

#### 2.4.1. Virtual Library

In a previous study on the antimalarial effects of TCLs [32], we have proposed a highly focused virtual combinatorial subset. Therefore, 40 smaller polar and larger aliphatic and aromatic hydrophobic R-groups (fragments, building blocks, reagents) were selected as suitable diverse building blocks for the position $R_{1}$. Out of them, 23 and 11 smaller aliphatic reagents were chosen as building blocks for the positions $R_{2}$ and $R_{3}$, respectively, which are known to be able to accommodate smaller substituents.

In this work, the virtual combinatorial subsets were extended to 60, 31 and 18 R-groups. The R-groups listed in Table 5 were attached to the positions $R_{1}$, $R_{2}$ and $R_{3}$ of the TCL scaffold to form a combinatorial library of the size: $R_{1}\times R_{2}\times R_{3}=60\times 31\times 18=33,480$ TCL analogues. This initial diversity library was generated from building blocks (chemicals) listed in the database of available chemicals [38].

#### 2.4.2. In Silico Screening

The generated virtual diversity library of 33,480 TCL analogues was further screened for molecular structures that matched the 3D-QSAR pharmacophore models of *Pf*ENR inhibition to identify compounds that are likely to bind to this target. During virtual screening, 1000 conformers were generated for each of 33,480 TCL analogues. After screening, 219 TCLs mapped to at least 2 pharmacophoric features of the 3D-QSAR pharmacophore model Hypo1 (Figure 5A,B) of inhibition of *Pf*ENR. These fitting analogues (PH4 hits) were then further evaluated by the complexation model. The computed relative GFE of the formation of the *Pf*ENR-TCLx complex, its components, and the predicted half-maximal inhibitory concentrations ${IC}_{50}^{pre}$ calculated from the correlation Equation (2) (Table 3) are listed in Table 6 (for a complete table, see Table S1, Supplementary Material).

### 2.5. Novel TCLs against PfENR

**Figure 6:**
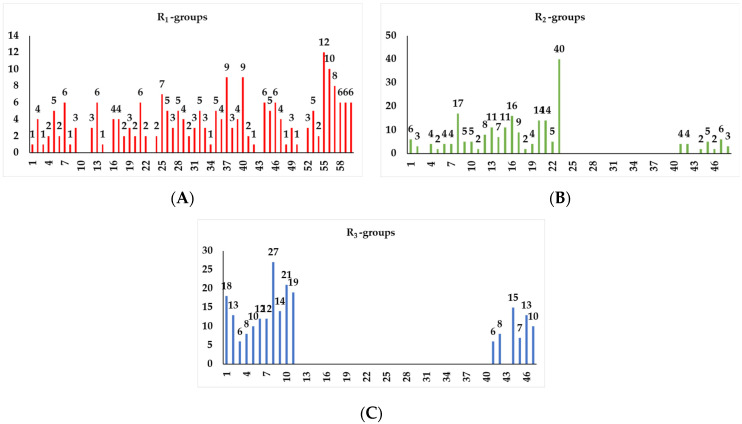
Histograms of the frequency of occurrence of individual fragments for the R-groups (**A**) R_1_, (**B**) R_2_, and (**C**) R_3_ in the 219 virtual TCL hits targeting *Pf*ENR.

The results of the in silico screening of the virtual library of novel TCL analogues were also analysed in terms of the frequency with which each individual fragment appears in the R groups of the 219 virtual hits that match the PH4 model of inhibition of *Pf*ENR. The R_1_ moiety of TCL that fills the hydrophobic pocket corresponding to the HYD pharmacophoric feature is a key determinant of improving the potency of TCLs against the *Pf*ENR. Five R_1_-groups emerged: No.**55**(*frequency*: *12*), **56**(*10*), **37**(*9*), **40**(*9*) and **59**(*8*) (Table 5, A). As we can see, these R_1_-groups include a linear heptyl chain or p-substituted phenyl ring connected to the TCL skeleton via a linker composed of 4 heavy atoms, similar to the **TCL11**. The $R_{2}$-groups are preferably occupied by medium-sized fragments such as: **23**(*40*), **8**(*17*), and **16**(*16*), while small-size R_3_-groups **8**(*27*), **10**(*21*), **11**(*19*) and **1**(*18*) contain chiefly small primary, secondary, and tertiary amines (Figure 6).

**Figure 7:**
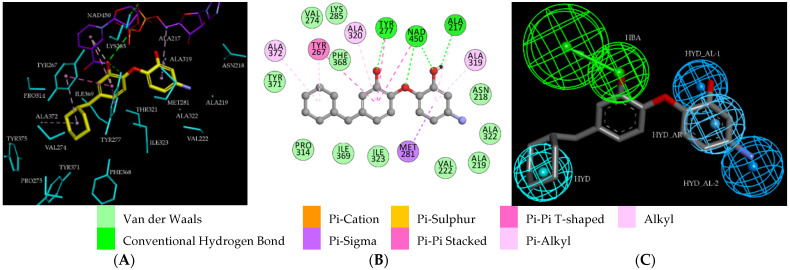
(**A**) Close-up of one of the most active designed TCL analogues, **TCL-33**-**08**-**05** (${IC}_{50}^{pre}$= 4.1 nM) at the active site of *Pf*ENR. The side chains of the interacting active site residues are coloured cyan, and carbon atoms of the ligand and the cofactor ${NAD}^{+}$ are colored yellow and purple, respectively. (**B**) 2D schematic interaction diagrams of the **TCL-33**-**08**-**05** at the active site of *Pf*ENR. Atom colouring: C-grey, N-blue, O-red. (**C**) Mapping of **TCL-33**-**08**-**05** to the pharmacophore model for *Pf*ENR.

Virtual TCL hits refined by molecular mechanics optimisation of the geometry of the *Pf*ENR-TCLx complexes include the analogues: **TCL-33**-**08**-**05** (${IC}_{50}^{pre}$ = 4.1 nM), **TCL-58**-**01**-**01** (${IC}_{50}^{pre}$ = 13.3 nM), **TCL-59**-**01**-**01** (${IC}_{50}^{pre}$ = 10.8 nM) **TCL-60**-**01**-**01** (${IC}_{50}^{pre}$ = 9.1 nM), **TCL-60**-**16**-**08** (${IC}_{50}^{pre}$ = 10 nM), **TCL-58**-**16**-**08** (${IC}_{50}^{pre}$ = 9.2 nM), **TCL-58**-**23**-**09** (${IC}_{50}^{pre}$ = 3.1 nM), and **TCL-60**-**08**-**08** (${IC}_{50}^{pre}$ = 1.9 nM) (Table 5 and Table 6). They include the following fragments at $R_{1}$-position: **33**: ···CH_2_-cyclohexyl; **58**: ···n-hexyl; **59**: ···n-heptyl; **60**: ···n-octyl. At $R_{2}$-position: 1: ···H; **8**: ···NH_3_^+^; **16**: ···trimethylamine; **23**: ···Ph; and at $R_{3}$-position **1**: ···H; **5**: ···OH; **8**: ···NH_3_^+^; **9**: ···C_2_H_5_. Due to the amino acid composition of the larger hydrophobic pocket, the $R_{1}$-groups display preferences for bulkier nonpolar building blocks of quite variable topology. The $R_{2}$-groups do not show a common character either, while the $R_{3}$-groups show preference mainly for small polar or cationic substituents. In the specific case of *Pf*ENR, the hydrophobic contacts are expected to root aryl-substituted inhibitors at the active site. The presence of small polar and cationic substituents in the $R_{3}$-position resulted in strong electrostatic attraction with the proximate phosphate groups of the cofactor nicotinamide adenine dinucleotide. The cationic substituents in the partially solvent-exposed $R_{3}$-position caused stabilization of the bound TCL analogues by the effect of hydration. These substitutions led to an overall predicted increase in the binding affinity of the new TCL analogues to *Pf*ENR. The best TCL analogues **TCL-60**-**08**-**08** and **TCL-33**-**08**-**05** (Figure 7) show predicted half-maximum inhibitory concentrations of (${IC}_{50}^{pre}$ = 1.9 nM and ${IC}_{50}^{pre}$ = 4.1 nM), i.e., could be almost 20 times more potent than the most active compound in the training set, namely **TCL15** (${IC}_{50}^{exp}=38nM$). Molecular mechanics refinement of the 219 top-ranking TCL analogues and prediction of the half-maximal inhibitory concentrations by means of computed rGFE of the ligands led to the identification of potent virtual hits with chemical structures that reach beyond the requirements of the PH4 pharmacophore model, especially in the $R_{2}$- and $R_{3}$-positions where hydrophobic interactions were expected.

**Figure 8:**
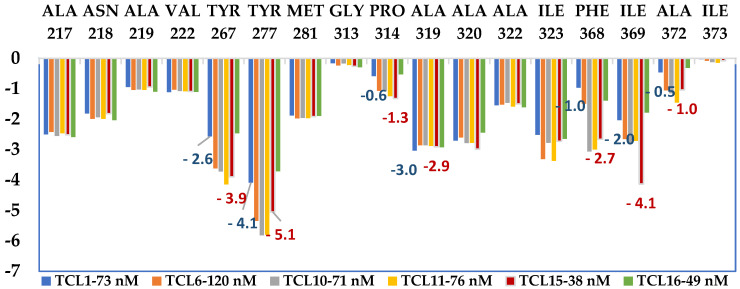
Plot of the energy of the intermolecular interaction $E_{int}$ between TCL inhibitors and *Pf*ENR, calculated by splitting the molecular mechanics into the contributions of individual residues for the most active TCL inhibitors, Table 1 [21,23,25,27], in [kcal·mol^−1^].

The role of hydrophobic residues of the active site pocket surrounding the R_1_-groups of TCLs is a key determinant of the predicted increase in the affinity of virtual hits. To gain a deeper understanding of enzyme-inhibitor interactions, the calculated interaction energy ($E_{int}$) of the most potent training set residues with the *Pf*ENR was analysed in terms of contributions from individual residues of the active site (Figure 8). To compare $E_{int}$ contributions of important active site residues to the total enzyme-inhibitor interaction energy for **TCL1** (${IC}_{50}^{exp}=73nM$) and **TCL15** (${IC}_{50}^{exp}=38nM$), the contributions of residues are listed: Tyr267 (−2.6 kcal·mol^−1^ for **TCL1** vs −3.9 kcal·mol^−1^ for **TCL15**), Tyr277 (−4.1 vs −5.1 kcal·mol^−1^), Gly313, Pro314 (−0.6 vs −1.3 kcal·mol^−1^), Phe368 (−1.0 vs −2.7 kcal·mol^−1^), Ile369 (−2.0 vs −4.1 kcal·mol^−1^), and Ala372 (−0.5 vs −1.0 kcal·mol^−1^). Taken together, this results in $E_{int}=$ −10.8 kcalvmol^−1^ for **TCL1** vs $E_{int}=$ −18.1 kcal·mol^−1^ for **TCL15**, approximately −7.3 kcal·mol^−1^ in favour of the more potent inhibitor **TCL15** (interactions with the cofactor were not considered). Analysis confirmed that the R_1_-groups contribute significantly to the inhibitor binding.

### 2.6. Pharmacokinetic Profile of Novel TCL Analogues

Properties related to ADME (absorption, distribution, metabolism, and excretion) were estimated for virtual hits, as well as for some selected reference antimalarial drugs. The best designed TCL derivatives with low oral bioavailability due to poor water solubility and rapid phase II metabolism must be disregarded for possible use as antimalarials. All of the ADME-related properties shown in Table 7, such as the octanol-water partition coefficient; aqueous solubility; blood-brain partition coefficient; Caco-2 cell permeability; serum protein binding; number of likely metabolic reactions; and another 18 descriptors of the new analogues, were computed by the QikProp program [39], based on the method of Jorgensen [40,41]. Experimental data from more than 710 compounds including about 500 drugs and related heterocycles were used to produce regression equations that correlated experimental and computed descriptors, resulting in a fast and accurate prediction of the pharmacokinetic properties of drug-like molecules. Consistent with the unfavourable pharmacokinetic characteristics observed for the best active triclosan derivatives, the predicted oral bioavailability of the new TCL analogues ranges from 86% to 100%. Since a value greater than 80% is considered good, six analogues among the best 10 hits show a human oral absorption in the gastrointestinal tract (HOA) of 100%. Drug-likeness (#stars)—the number of property descriptors that fall outside the optimal value range determined for 95% of known drugs out of 24 selected descriptors computed by QikProp—was used as an additional general criterion for selecting compounds with good ADME-related properties. The values of the best active designed TCLs against *Pf*ENR are compared with those computed for drugs used for the treatment of malaria alone or, in the case of artemisinin, drugs used in a combined therapy (ACT), or drugs currently undergoing clinical trials (Table 7). This comparison revealed that triclosan analogues are endowed with ADME-related properties which are comparable to most of the considered antimalarial drugs.

### 2.7. Molecular Dynamics Simulations

**Figure 9:**
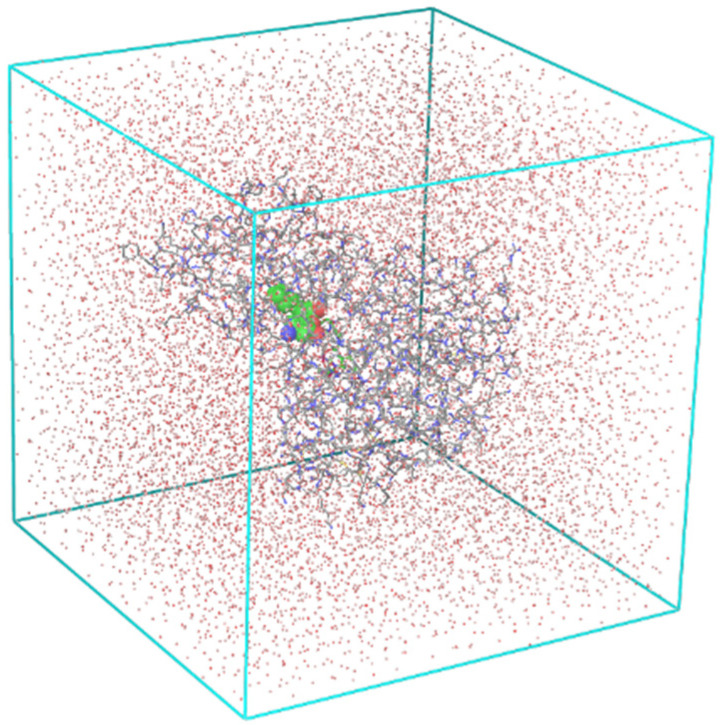
The periodic cubic box used in MD simulations contains the solvated *Pf*ENR-TCL complex.

**Figure 10:**
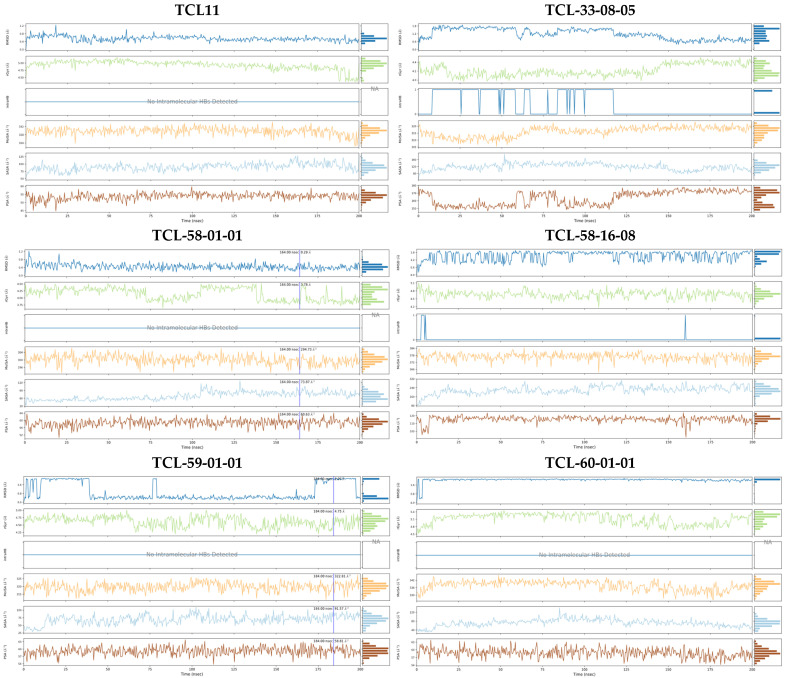
Time-evolution of the properties of the six *Pf*ENR-TCLx complexes during 200 ns MD simulation. For each inhibitor, top to bottom: plot of the root mean square deviation (RMSD) with respect to the initial conformation vs. the simulation time, radius of gyration (rGyr), number of intramolecular hydrogen bonds (intraHB), molecular surface area (molSA), solvent-accessible surface area (SASA), and polar surface area (PSA).

**Figure 11:**
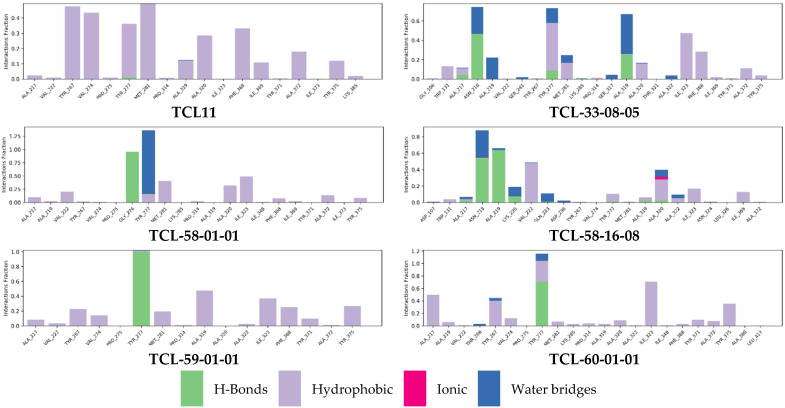
Contribution of individual active site residues to inhibitor binding in *Pf*ENR-TCLx complexes present during MD simulations: HB (green); ionic interactions (magenta); hydrophobic contacts (purple); water bridges (blue).

**Figure 12:**
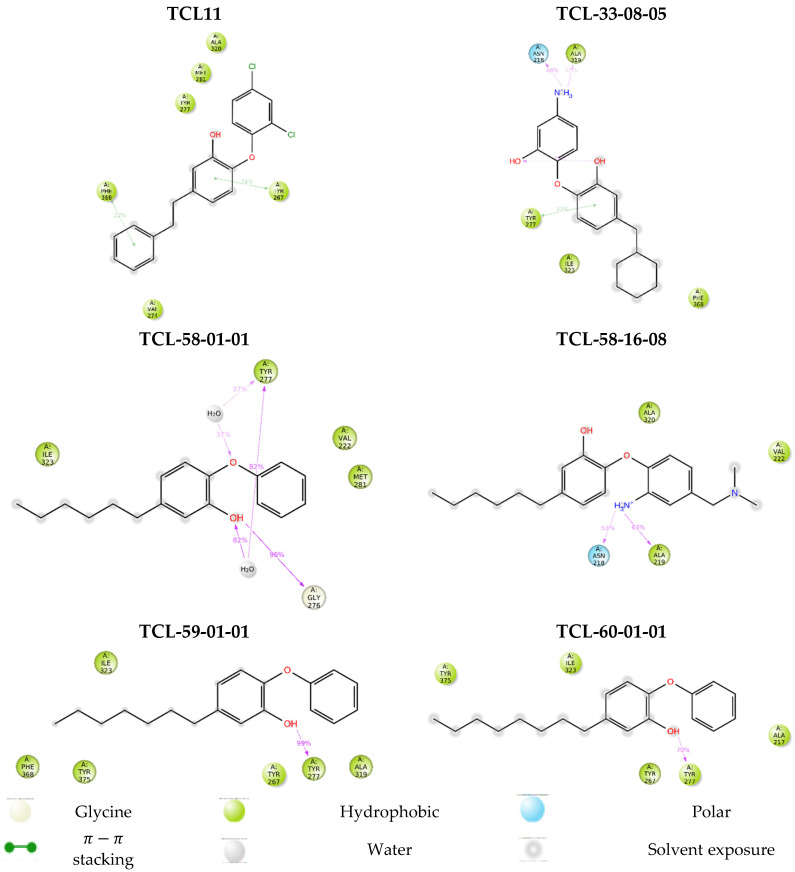
2D representation of the most populated attractive interactions between the function groups of the six inhibitors and the individual residues at the active site of *Pf*ENR that occur in at least in 1/5 of the 500 analysed frames.

**Figure 13:**
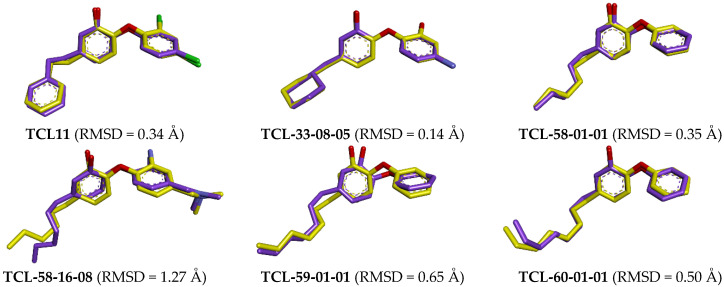
Superimposition of the ligand active conformation from complexes refined by molecular mechanics (yellow carbons, red oxygens, blue nitrogens) and averaged active conformations resulting from MD simulations (purple carbon atoms).

We also performed molecular dynamics simulations to check the stability of *Pf*ENR-inhibitor complexes and the flexibility of the active conformations of the native ligand **TCL11** and five of the best-designed TCL analogues at the active site of *Pf*ENR. Thus, the complexes obtained from the modification in situ of the reference inhibitor **TCL11** and subsequent refinement through molecular mechanics were used as starting geometries for the molecular dynamics calculations using the Desmond software [42] (see Section 4). Table 8 presents the averages of total energy $\langle E_{tot}\rangle$ and potential energy $\langle E_{pot}\rangle$ of the systems during the 200 ns simulation. Figure 9 illustrates the simulated system and Figure 10 shows the time evolution of bound inhibitor properties, such as the root mean square deviation (RMSD) from the initial conformation, the radius of gyration (rGyr), number of intramolecular hydrogen bonds (intraHB) molecular surface area (molSA), solvent-accessible surface area (SASA) and the polar surface area (PSA). Protein-ligand interactions were explored throughout the simulation trajectory to identify specific interactions maintained during computation (Figure 11). Finally, the interactions that occur more than 20.0% of the simulation time in the trajectory (0–200 ns) are shown on a detailed 2D schematic representation (Figure 12). Moreover, we have superimposed the conformations of the ligands obtained following the minimisation of the complexes from molecular dynamics on those obtained by in situ modification of the **TCL11** and MM refinement. Figure 13 illustrates these superimpositions and the respective RMSDs. The RMSD, rGyr, SASA, and the remaining parameters document that the pose of the bound new TCLs in the complexes with *Pf*ENR remains stable within the MD simulation time (Figure 10). The higher contribution of hydrogen bonding interactions of the new TCL analogues with the active site residues as compared to the predominantly hydrophobic interaction of the **TCL11** reference compound suggests an elevated specificity of these virtual hits for the *Pf*ENR target (Figure 11).

## 3. Discussion

### 3.1. Binding Mode of TCL

Freundlich et al. reported the most comprehensive study of inhibition of *PfENR* by triclosan (**TCL1**) [21,23,27]. Characteristic intermolecular interactions of **TCL1** and *Pf*ENR include 𝜋-𝜋 stacking interaction between the phenol moiety and the nicotinamide ring of ${NAD}^{+}$, hydrogen bonds between the hydroxyl group of **TCL1** and -OH group of Tyr277 and the C_3_-OH group of the ribose ring of ${NAD}^{+}$. According to our analysis of the *Pf*ENR-TCLx complexes of predicted most potent inhibitors, these key interactions are conserved throughout this class of compounds and contribute also to predicted inhibitory potencies of novel triclosan derivatives. In addition, in the $R_{3}$-position of triclosan, a H-bond with the backbone of residue Ala217 and with the ${NAD}^{+}$ cofactor was reported by Kumar et al. [31] (Figure 7). The most potent analogues **TCL-60-08-08** and **TCL-33-08-05** with the $R_{1}$-group ···CH_2_-cyclohexyl or the flexible ···octyl do not have the capacity to form stacking interactions but can still well fill the hydrophobic pocket formed by residues Tyr267, Pro314, Ile369, Phe368, and Ala372. These $R_{1}$-groups, in combination with protonated amine or hydroxyl groups in the $R_{2}$-position and $R_{3}$-position that interact with the phosphate groups of ${NAD}^{+}$ or with solvent, also contribute substantially to stabilisation of the *Pf*ENR-TCLx complexes. Thus, new TCL derivatives with potential to be developed into novel antimalarial agents that act on a new pharmacological target are proposed to medicinal chemistry laboratories for experimental verification.

### 3.2. Molecular Dynamics Simulations

Low deviations between the superimposition of active conformations of TCL inhibitors modelled by in situ modification of **TCL11** and MM refinement and those obtained as ensemble averages over 500 snapshots from MD trajectories (Figure 13), suggest that the modelled complexes *Pf*ENR-TCLx are stable. Moreover, MD simulations confirmed the validity of the binding mode of new triclosan analogues generated from the crystal structure of *Pf*ENR-**TCL11** [21].

## 4. Materials and Methods

**Scheme 1:**
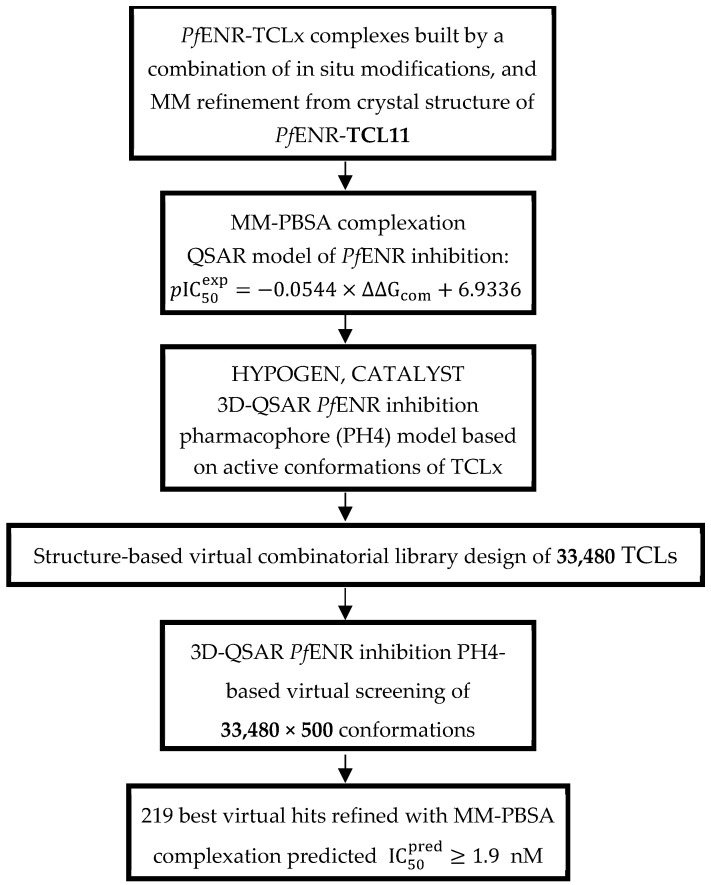
Workflow describing the sequence of steps taken to virtually design novel TCL analogues with higher predicted potencies against *Pf*ENR.

The workflow describing consecutive steps of the combinatorial ligand design and the in silico process of novel TCL analogues against *Pf*ENR is presented in Scheme 1.

### 4.1. Training and Validation Sets of TCL Inhibitors

The chemical structures and biological activities (${IC}_{50}^{exp}$) of training and validation sets of triclosan (TCL) based inhibitors of *Pf*ENR used in this study were taken from the literature [21,23,25,27]. The inhibitory potencies of these compounds cover a sufficiently broad range of half-maximal inhibitory concentrations ($38\;nM\leq {IC}_{50}^{exp}\leq 10^{5}nM$) to allow construction of a QSAR model. The training set (TS) contains 20 triclosan derivatives and the validation set (VS) contains 4 triclosan derivatives, taken from the refs. [21,23,25,27].

### 4.2. Model Building

Molecular models of the enzyme-inhibitor complexes *Pf*ENR-TCLx (E–I), free enzyme *Pf*ENR (E), and free inhibitors (I) were prepared from the high-resolution crystal structure of the reference complex *Pf*ENR-**TCL11** (Protein Data Bank [43] entry code 2OOS at a resolution of 2.1 Å [21]) using Insight-II molecular modelling program [44]. The structures of the E and E–I complexes were considered at an appropriate physiologically relevant pH of 7 with capped neutral N- and C-terminal residues and all protonated and ionised residues charged. Crystallographic water molecules were included in the model. TS and VS were built into the structure of the reference complex *Pf*ENR-**TCL11** by in situ replacement of the derivatised R groups of the TCL moiety. An exhaustive conformational search over all rotatable bonds of the replacing function group, coupled with a careful gradual energy optimisation of the modified inhibitor and the *Pf*ENR active site residues located in the vicinity of the inhibitor (within 5 Å distance), was employed to identify the low energy bound conformations of the modified inhibitor. The resulting low-energy structures of the E–I complexes were then carefully refined by minimisation of the whole protein. This procedure has previously been successfully used for model building of viral, bacterial, and protozoal protease inhibitor complex models and design of peptidomimetic, hydroxy naphthoic, and thymidine-based inhibitors [45,46,47,48,49,50].

### 4.3. Molecular Mechanics

The modelling of inhibitors, the *Pf*ENR and E–I complexes was carried out by molecular mechanics (MM) as previously described [47]. The TCL inhibitors, the *Pf*ENR enzyme, and their complex (E–I) were modelled in all-atom representation (parameters and charges) with the CFF force field. During all MM calculations, the dielectric constant was set to 4 to consider the dielectric shielding effect in proteins. The geometry optimisation process was performed gradually by relaxing the molecular structure through a sufficient number of steepest descent steps followed by iterative conjugate gradient cycles to reach the convergence criterion of 0.01 kcal·mol^−1^·Å^−1^ for the average gradient.

### 4.4. Conformational Search

The free inhibitor conformations were derived from their bound configurations in the E–I complexes by gradual relaxation to the nearest local energy minimum as previously described [46,47,48]. Monte Carlo search was carried out (for 50,000 iterations) over all rotatable bonds (excluding the rings) using Discovery Studio [34]. Two hundred unique stable conformations were generated for each TCL by random variation of the torsion angles of the last accepted conformer by ±15° at 5000 K before energy minimisation with a dielectric constant ε = 80 to account for the dielectric screening effect of solvation. The lowest energy conformer was selected and minimised again at a dielectric constant of 4.

### 4.5. Solvation Gibbs Free Energies

The electrostatic component of Gibbs free energy (GFE) [50] that includes the effects of ionic strength by solving the nonlinear Poisson–Boltzmann equation [51,52] was computed by the DelPhi module in Discovery Studio [34] as previously described [46]. The implicit model of solvation represents the solvent by a continuous medium of high dielectric constant (ε_o_ = 80) and the solute as charge distribution filling a low dielectric (ε_i_ = 4) cavity with boundaries related to the molecular surface. The module numerically solves for the molecular electrostatic potential and reaction field around the solute using the finite difference method. DelPhi calculations were done on a (235 × 235 × 235) cubic lattice grid for the E–I complexes and free E and on a (65 × 65 × 65) grid for the free I. Full coulombic boundary conditions were used. Two subsequent focussing steps led to a similar final resolution of about 0.3 per grid unit at 70% filling of the grid by the solute. Physiological ionic strength of 0.145 mol·dm^−3^, partial atomic charges and radii defined in the set of CFF force field parameters [34] and a probe sphere radius of 1.4 were used. The electrostatic component of the Poisson–Boltzmann solvation Gibbs free energy was calculated as the reaction field energy [53,54,55].

### 4.6. Calculation of the Entropic Contribution

The changes in vibrational entropy during the binding of the inhibitor to the enzyme (E) were calculated by the normal mode analysis of inhibitor vibrations using a simplified method based on Fischer et al. [56]. In this approach, vibrational analyses of the inhibitor bound at the active site of a ‘frozen’ E and of the low energy conformer of the free inhibitor are calculated for fully minimised structures.

### 4.7. Calculation of Binding Affinity and QSAR Model

The calculation of the binding affinity expressed as rGFE of complex formation has been fully described earlier [46,47,48].

### 4.8. Interaction Energy

The calculation of MM interaction energy (E_int_) between enzyme residues and the inhibitor was performed as previously described [47,48].

### 4.9. Pharmacophore (PH4) Generation

According to the International Union of Pure and Applied Chemistry, a pharmacophore is “an ensemble of steric and electronic features that is necessary to ensure optimal supramolecular interactions with a specific biological target structure and to trigger (or block) its biological response” [57]. The evolution of the concept of pharmacophore in computer-assisted drug design has previously been previously reported [58,59,60]. The main task is to identify the highest spatial arrangement of common structural features shared by a group of molecules, making them recognisable by the active site of the biomolecular target and therefore characterising the affinity towards that target and consequently its biological activity. The first goal of the pharmacophore (PH4) is to estimate biological activity. Since the 3D Quantitative Structure Activity Relationships (3D-QSAR) Pharmacophore built here takes account of inhibitor–target interaction patterns, it is referred to as a structure-based model (differently from the model generated from a set of ligands without involving the target).

In this work, 3D-QSAR pharmacophore models were built using the Catalyst HypoGen algorithm [61] implemented in Discovery Studio [34] from the bound bioactive conformations of inhibitors taken from the QSAR models of E–I complexes. The E–I complexes were derived from the high resolution crystal structure of *Pf*ENR-**TCL11** [21], so no new bioactive TCL conformations were generated during the process. Kurogi and Güner reported in a review the complete theory and successful applications of the PH4 generation process using the Catalyst software [62]. On the basis of the biological activities, HypoGen will optimise the pharmacophore hypotheses deduced from the training set inhibitors with the highest activity and omitting the least active molecules. The top scoring hypothesis of the pharmacophore will be built up in three main stages (constructive, subtractive and optimisation steps) from the set of most active inhibitors [62]. Inactive molecules serve for the definition of the excluded volume. The maximum number of five features allowed by the HypoGen algorithm was selected on the basis of the TCL scaffold and substituents during the pharmacophore generation, namely: hydrophobic aromatic (HYdAr), hydrophobic aliphatic (HYd), hydrogen-bond donor (HBD), hydrogen-bond acceptor (HBA) and ring aromatic (Ar). Many remaining features, among which positive charge (PC), positive ionisable (PIC) and negative charge (NC), negative ionisable (NIC) were not selected because of the neutral type of TCL molecules considered in this study. Additional features may be added if necessary [60]. Today, the concept of pharmacophore has been extended beyond the initial horizon of drug design to reach chemoinformatics and material-informatics QSPR as “Photovoltaphores” to identify metal-free dyes for dye-sensitized solar cells [63]. The adjustable parameters of the protocol were kept at their default values, except for the uncertainty of the activity, which was set to 1.25 instead of 3. Uncertainty is the range of the activity value with a default of <IC_50_/3, 3 IC_50_>. Our choice to bring the uncertainty interval for experimental activity to a relatively narrow <4 IC_50_/5, 5 IC_50_/4> takes into account the precision and homogeneity of the observed inhibitory activities measured by the same method in a single laboratory. Therefore, during the constructive step, the activity of the most active (MA) compound is multiplied by 1.25 instead of 3 to reach a value A = $\frac{{5\times IC}_{50}(MA)}{4}$ and the activity of the second most active (NMA) is multiplied by 4/5 to give a value B = $\frac{{4\times IC}_{50}(NMA)}{5}$. In case B is smaller than A, NMA is included in the set of most active compounds. Assuming the set of default values of 3.0 A’ = ${3\times IC}_{50}(MA)$ and B’ = $\frac{{1\times IC}_{50}(NMA)}{3}$ broadens the most active training set excessively. The subtractive step will consider as inactive those TCL compounds with activity 3.5 orders of magnitude beyond ${IC}_{50}(MA)$: ${IC}_{50}=10^{3.5}{IC}_{50}(MA)$. Any pharmacophore that matches more than half of the set of inactive TCLs is removed. The default value of 3.5 is user adjustable if the range of experimental activities does not span 3.5 orders of magnitude [62]. For the optimisation phase, the hypothesis score will be improved for the remaining pharmacophore configurations by perturbations based on the estimated errors of the activity during the linear regression and complexity of each hypothesis. According to the law of parsimony (Occam’s razor) “the simpler hypothesis that accurately estimates the activity is considered the best” [62].

During the generation of 10 pharmacophores, the number of missing features was set to 0 and the best pharmacophore was selected. The reliability of the generated pharmacophore models was assessed on the calculated cost parameters (in units of the number of bits required to generate them). The overall cost (or total cost) of a model is the sum of three components: (i) the weight, (ii) the error, and (iii) the configuration cost:

$$Total\;cost=Cost\left(error\right)\times CC\left(error\right)+Cost\left(weight\right)\times CC\left(weight\right)+Cost\left(ConFigure\right)\times CC\left(ConFigure\right)$$

where CCs are cost coefficients in the Catalyst module of Discovery Studio [34]. $Cost\left(error\right)$ and $Cost\left(weight\right)$ are estimated through a Gaussian probability distribution:

- Cost(error) increases as the Root Mean Square difference between the estimated and experimental activities for the training set compounds;
- $Cost\left(weight\right)$ increases in a Gaussian form as the feature weight deviates from an ideal value (2.0);
- The configuration cost $Cost\left(ConFigure\right)$ is a fixed cost depending on the complexity of the hypothesis space being optimised. It is equal to the entropy of the hypothesis space (log_2_P, P: the number of hypotheses initially created in a constructive phase that emerged through the subtractive one). In the standard HypoGen mode, its value should not exceed 17.

Details on costs and score calculations can be found in [49]. Two additional costs are calculated: the fixed cost and the null cost during the generation of the pharmacophore. They are theoretical costs helping to appreciate the significance of each PH4 hypothesis, since the closer the cost is to the fixed cost and the farther it is from the null cost the more significant it is statistically and the more likely to be a true correlation rather than a chance correlation.

Fixed cost represents the lowest possible cost of the perfect pharmacophore model that ideally fits the data (lower bound). Fixed costs are calculated by summing up the minimum possible error and weight cost plus the constant configuration cost. The null cost is another cost parameter; it stands for the maximum cost of a pharmacophore with no features and corresponds to the averaged activity data of the training set molecules (upper bound).

### 4.10. ADME Properties

Properties that determine the pharmacokinetic profile of a compound, in addition to the coefficient of octanol/water partitioning, aqueous solubility, blood/brain partition coefficient, Caco-2 cell permeability, serum protein binding, number of likely metabolic reactions and 18 more descriptors related to adsorption, distribution, metabolism and excretion (ADME properties) of the inhibitors, were computed by the QikProp programme [39] based on the methods of Jorgensen [40,41]. According to these methods, experimental results of more than 710 compounds were correlated with computed physicochemical descriptors, which resulted in an accurate prediction of the pharmacokinetic profile of considered new molecules. Drug likeness (#stars)—the number of property descriptors that fall outside the range of values determined for 95% of known drugs out of 24 selected descriptors computed by QikProp [39], was used as an additional ADME-related compound selection criterion.

### 4.11. Virtual Library Generation

The virtual library generation was performed as previously described [47]. Analogue model building was performed with the Molecular Operating Environment (MOE) programme [64]. The library of analogues was enumerated by attaching R groups (fragments, building blocks) to the MOE TCL scaffold using the Quasar CombiDesign module [64]. Chemical reagents considered in this study were taken from the directories of chemicals available from the commercial suppliers of chemicals [38]. Each analogue was built as a neutral molecule in the MOE program and its molecular geometry was refined by MM optimization using smart minimizer of Discovery Studio [34] with high convergence criteria (energy difference of 10^−4^ kcal·mol^−1^, R.M.S. displacement of 10^−5^ Å) and a dielectric constant of 4 using class II consistent force field CFF [34] as described in Section 4.3.

### 4.12. ADME-Based Library Focussing

The drug-likeness selection criterion served to focus the initial virtual library as previously described [65].

### 4.13. Pharmacophore-Based Library Searching

The pharmacophore model (PH4) described in Section 4.8 and derived from the bound conformations of TCLs at the active site of *Pf*ENR served as a library search tool and in silico screening instrument as previously described [35]. The enumerated virtual library was further focused by using the ligand pharmacophore mapping protocol available from Discovery Studio [34]. Within this protocol, each generated conformer of the analogues was geometry optimised by means of the CFF force field for a maximum of 500 energy minimisation steps and subsequently aligned and mapped to the PH4 model to select the top-ranking overlaps. Twenty best-fitting conformers were saved and clustered into ten conformational families according to their mutual r.m.s.d by means of the Jarvis–Patrick complete linkage clustering method [66]. The best representative of each cluster was considered in the virtual screening of analogues.

### 4.14. Inhibitory Potency Prediction

The conformer with the best mapping on the PH4 pharmacophore in each cluster of the focused library subset was used for ${∆∆G}_{com}$ calculation and ${IC}_{50}^{pre}$ estimation by the complexation QSAR model as previously described [49]. The rGFE of formation of the E–I complex in water ${∆∆G}_{com}$ was computed for each selected new analogue and then used to predict the *Pf*ENR inhibitory potencies (${IC}_{50}^{exp}$) of the focused virtual library of TCL analogues by inserting this parameter into the target-specific scoring function, Equation (2), Table 3. The scoring function, which is specific for the *Pf*ENR receptor: ${pIC}_{50}^{exp}=a\times {∆∆G}_{com}+b$, was parameterised using the QSAR model described in Section 4.7.

### 4.15. Molecular Dynamics Simulations

The conformational stability of the co-crystallised ligand (**TCL11**) and the five selected top-ranking designed TCL analogues (**TCL-33-08-05**, **TCL-58-01-01**, **TCL-58-16-08**, **TCL-59-01-01**, **TCL-60-01-01** obtained by in situ modification of the reference inhibitor **TCL11** and refinement was evaluated by molecular dynamics (MD) simulations as described recently [67]. We have carried out 200 ns long simulations of these five explicitly solvated complexes in the NPT statistical ensemble (300 K, 1 bar) by using Desmond software [42]. A cubic periodic box with 10 Å buffer containing the E–I complex was filled with TIP3P water molecules and neutralised by adding the required number of counterions to reach electroneutrality (Table 9). During the simulation, OPLS4 force field [68], 1.5 fs integration step, and the coulombic interaction cut-off point of 14 Å, were used. The Nose–Hoover chains thermostat and Martyna–Tobias–Klein barostat methods were employed during the simulations [69,70]. After initial heating and relaxation, the productive simulation trajectory was recorded and analysed for ligand-receptor interactions every 400 ps, that is, 500 frames during each MD simulation.

After MD calculations, the lowest total energy frame of each complex *Pf*ENR-inhibitor was minimised by molecular mechanics in 4 steps (as previously described [32]) using the OPLS4 force field in the Schrodinger software [42].

## 5. Conclusions

In this work, novel derivatives of TCL inhibiting ENR of *Plasmodium falciparum* that reach low nanomolar (one digit) inhibitory concentrations ${IC}_{50}^{pre}$ have been designed (Table 8, Figure 12). A 3D-QSAR-based pharmacophore model of *Pf*ENR inhibition by a training set of 20 triclosan derivatives was used for in silico screening of a virtual combinatorial library of more than 33,000 triclosan analogues. More than 200 best virtual hits were modelled at the active site of *Pf*ENR using the crystal structure of *Pf*ENR-**TCL11** complex [21] and their inhibitory potencies toward *Pf*ENR were predicted from computed rGFE of E–I complex formation, which correlated with the observed activities ${IC}_{50}^{exp}$ of the training and validation set of TCLs. In silico screening, refinement of the structure of *Pf*ENR-TCLx complexes and prediction of ${IC}_{50}^{pre}$ enabled identification of new TCL analogues with optimal substitutions in the $R_{1}$, $R_{2},$ and $R_{3}$-positions of the TCL scaffold (Table 1). New triclosan analogues with predicted inhibitory potencies **TCL-60-08-08** (${IC}_{50}^{pre}$ = 1.9 nM), **TCL-33-08-05** (${IC}_{50}^{pre}$ = 4.1 nM), and **TCL-33-16-08** with (${IC}_{50}^{pre}$= 7.8 nM) display high predicted binding affinity to *Pf*ENR (Table 6), and favourable ADME-related properties (Table 7). Therefore, they could form new prospective candidates for reversible inhibitors aimed at the new validated pharmacological target of *P. falciparum* so far not connected with drug resistance to clinically used antimalarial therapeutics. Molecular dynamics simulations confirmed the stability of these enzyme–inhibitor complexes. Therefore, we can recommend these new molecules for synthesis and experimental testing of their inhibitory potency and antimalarial effect on the *Pf*ENR.

## Figures and Tables

**Table 1:** Training and validation sets of TCL inhibitors used for the preparation of QSAR models of *Pf*ENR inhibition. The experimental activities were taken from [21,23,25,27].

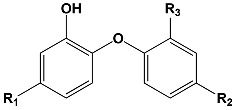				
Training Set (TS)	$R_{1}$	$R_{2}$	$R_{3}$	${IC}_{50}^{exp}$ [nM]
TCL1 (Triclosan)	⋯Cl	⋯Cl	⋯Cl	73
TCL2	$\cdots {CH}_{3}$	⋯Cl	⋯Cl	200
TCL3	${\cdots CH}_{2}CycHex$	⋯Cl	⋯Cl	530
TCL4	$\cdots {\left({CH}_{2}\right)}_{3}{CH}_{3}$	⋯Cl	⋯Cl	480
TCL5	$\cdots {CH}_{2}CH{\left({CH}_{3}\right)}_{2}$	⋯Cl	⋯Cl	180
TCL6	$\cdots {\left({CH}_{2}\right)}_{2}CH{\left({CH}_{3}\right)}_{2}$	⋯Cl	⋯Cl	120
TCL7	$\cdots {CH}_{2}CH\left({CH}_{3}\right){CH}_{2}{CH}_{3}$	⋯Cl	⋯Cl	290
TCL8	$\cdots {CH}_{2}-2-Pyr$	⋯Cl	⋯Cl	640
TCL9	$\cdots {CH}_{2}-4-Pyr$	⋯CN	⋯Cl	530
TCL10	$\cdots {CH}_{2}-Ph$	⋯Cl	⋯Cl	71
TCL11	$\cdots {\left({CH}_{2}\right)}_{2}-Ph$	⋯Cl	⋯Cl	76
TCL12	⋯Ph	⋯Cl	⋯Cl	140
TCL13	$\cdots Ph-o{CH}_{3}$	⋯Cl	⋯Cl	440
TCL14	$\cdots Ph-o{CH}_{3}$	⋯CN	⋯Cl	410
TCL15	⋯Ph−pF	⋯Cl	⋯Cl	38
TCL16	⋯CN	⋯Cl	⋯Cl	49
TCL17	$\cdots Ph-p{CH}_{3}$	⋯Cl	⋯Cl	190
TCL18	… Cl	⋯H	…NH_2_	7000
TCL19	…2H-tetrazol-5-yl	… Cl	… Cl	100,000
TCL20	… 3-pyridyl	…Cl	…Cl	33,000
Validation Set (VS)	$R_{1}$	$R_{2}$	$R_{3}$	${IC}_{50}^{exp}$ [nM]
TCL21	$\cdots {\left({CH}_{2}\right)}_{2}{CH}_{3}$	⋯Cl	⋯Cl	210
TCL22	$\cdots {CH}_{2}{CH}_{3}$	⋯Cl	⋯Cl	110
TCL23	$\cdots {CH}_{2}-\left(3-Pyr\right)$	⋯Cl	⋯Cl	840
TCL24	$\cdots Ph-m{CH}_{3}$	⋯Cl	⋯Cl	230

**Table 2:** Relative complexation Gibbs free energy (binding affinity, rGFE) and its components for the training set of the *Pf*ENR inhibitors **TCL1–20** and the validation set inhibitors **TCL21–24**.

Training Set ^a^	${∆∆H}_{MM}$ ^b^ ${[kcal\cdot mol}^{-1}]$	${∆∆G}_{sol}$ ^c^ ${[kcal\cdot mol}^{-1}]$	${∆∆TS}_{vib}$ ^d^ ${[kcal\cdot mol}^{-1}]$	${∆∆G}_{com}$ ^e^ ${[kcal\cdot mol}^{-1}]$	${IC}_{50}^{exp}$ ^f, g^ [nM]
TCL1	0	0	0	0	73
TCL2	8.88	−2.30	2.06	4.52	200
TCL3	15.58	−0.16	5.38	10.04	530
TCL4	14.87	−2.65	3.90	8.31	480
TCL5	11.16	−2.60	4.16	4.40	180
TCL6	6.35	−0.94	4.90	0.51	120
TCL7	13.45	−2.34	4.53	6.58	290
TCL8	8.68	0.12	0.62	8.17	640
TCL9	8.21	−0.20	2.86	5.15	530
TCL10	0.11	0.62	2.38	−1.65	71
TCL11	2.71	0.19	2.25	0.66	76
TCL12	4.01	0.03	2.99	1.04	140
TCL13	11.64	−1.93	1.69	8.02	440
TCL14	12.08	−1.21	4.18	6.69	410
TCL15	−0.78	−0.73	1.81	−3.33	38
TCL16	−1.38	−1.61	0.46	−3.44	49
TCL17	2.97	−0.45	−0.04	2.56	190
TCL18	31.80	6.56	2.24	36.13	7 000
TCL19	55.03	−0.34	1.58	53.11	100,000
TCL20	37.93	12.97	3.3	47.61	33,000
Validation Set ^a^	${∆∆H}_{MM}$ ^b^ ${[kcal\cdot mol}^{-1}]$	${∆∆G}_{sol}$ ^c^ ${[kcal\cdot mol}^{-1}]$	${∆∆TS}_{vib}$ ^d^ $\left[{kcal\cdot mol}^{-1}\right]$	${∆∆G}_{com}$ ^e^ ${[kcal\cdot mol}^{-1}]$	${pIC}_{50}^{pre}/{pIC}_{50}^{exp}$ ^f^
TCL21	10.47	−1.95	3.94	4.75	1.00
TCL22	7.53	−1.41	3.80	2.32	1.02
TCL23	7.51	0.89	1.14	7.26	0.93
TCL24	7.17	−1.19	2.29	3.70	0.99

^a^ for chemical structures of the triclosan analogues of TS and VS, see Table 1; ^b^ ${∆∆H}_{MM}$ is the relative enthalpic contribution to the change in rGFE in the formation of the enzyme-inhibitor E:I complex formation derived from molecular mechanics (MM): ${∆∆H}_{MM}≅\left[E_{MM}\left\{E:I_{x}\right\}-E_{MM}\left\{I_{x}\right\}\right]-\left[E_{MM}\left\{E:I_{ref}\right\}-E_{MM}\left\{I_{ref}\right\}\right]$, $I_{ref}$ is the reference inhibitor **TCL1**; ^c^ ${∆∆G}_{sol}$ is the solvent effect contribution to the rGFE change of the E:I complex formation: ${∆∆G}_{sol}=\left[G_{sol}\left\{E:I_{x}\right\}-G_{sol}\left\{I_{x}\right\}\right]-\left[G_{sol}\left\{E:I_{ref}\right\}-G_{sol}\left\{I_{ref}\right\}\right]$; ^d^ ${-∆∆TS}_{vib}$ is the relative entropic contribution to the rGFE of E:I complex formation: ${∆∆TS}_{vib}=\left[{TS}_{vib}{\left\{I_{x}\right\}}_{E}-{TS}_{vib}\left\{I_{x}\right\}\right]-\left[{TS}_{vib}{\left\{I_{ref}\right\}}_{E}-{TS}_{vib}\left\{I_{ref}\right\}\right]$; ^e^ ${∆∆G}_{com}$ is the rGFE change of E:I complex formation: ${∆∆G}_{com}≅{∆∆H}_{MM}+{∆∆G}_{sol}-{∆∆TS}_{vib}$. ^f^ ${IC}_{50}^{exp}$ is the experimental half-maximal inhibitory concentration of TCLs against *Pf*ENR obtained from the literature [22,24,26,28]. ^g^ The ratio of predicted and experimental half-maximal inhibitory concentrations ${pIC}_{50}^{pre}/{pIC}_{50}^{exp}$. ${pIC}_{50}^{pre}=-\log_{10}{IC}_{50}^{pre}$ was predicted from the computed ${∆∆G}_{com}$ using the regression Equation (2) for *Pf*ENR shown in Table 3.

**Table 3:** Regression analysis of computed binding affinities ${∆∆G}_{com}$, its enthalpic component ${∆∆H}_{MM}$, and experimental half-maximal inhibitory concentrations ${pIC}_{50}^{exp}=-\log_{10}{IC}_{50}^{exp}$ of TCLs towards the *Pf*ENR [21,23,25,27].

Statistical Data of Regression	(1)	$\left(2\right)$
${pIC}_{50}^{exp}=-0.0614\times {∆∆H}_{MM}+7.1496\;\;(1)$		
${pIC}_{50}^{exp}=-0.0544\times {∆∆G}_{com}+6.9336\;\;(2)$		
Number of compounds in TS	20	20
Squared correlation coefficient of regression $R^{2}$	0.94	0.95
Cross-validated squared correlation coefficient $R_{xv}^{2}$	0.92	0.93
Standard error of regression σ	0.21	0.18
Statistical significance of regression, Fisher F-test	327.3	426.16
Level of statistical significance α	>95%	>95%
Range of experimental activities ${IC}_{50}^{exp}$ [nM]	38–100,000	

**Table 4:** Parameters of 10 generated PH4 pharmacophoric hypotheses for the *Pf*ENR inhibitor after the CatScramble validation procedure (49 scrambled runs for each hypothesis at the selected confidence level of 98%).

Hypothesis	RMSD ^a^	$R^{2}$ ^b^	Total Cost ^c^	Costs Difference ^d^	Closest Random ^e^	Features ^f^
Hypo1	6.565	0.96	409.3	5013.9	1021.7	HBA, HYD-AL, HYD-AL, HYD-Ar, HYD
Hypo2	9.310	0.93	803.5	4619.7	1035.5	HBA, HYD-AL, HYD-AL, HYD, HYD
Hypo3	9.428	0.92	822.7	4600.5	1058.9	HBA, HYD-AL, HYD-Ar, HYD, HYD
Hypo4	9.587	0.92	850.3	4572.9	1068.1	HBA, HYD-AL, HYD, HYD, HYD
Hypo5	10.776	0.90	1069.8	4353.4	1076.6	HYD-AL, HYD-AL, HYD-Ar, HYD-Ar, HYD
Hypo6	10.780	0.90	1071.3	4352.0	1079.7	HYD-AL, HYD-AL, HYD-Ar, HY, HY
Hypo7	18.776	0.64	3195.9	2227.3	1088.2	HYD-AL, HYD-Ar, HYD-Ar, HY, HY
Hypo8	11.420	0.89	1195.3	4227.9	1090.9	HBA, HYD-AL, HY, HYD, HYD
Hypo9	18.835	0.64	3216.9	2206.4	1099.1	HYD-AL, HYD-Ar, HYD, HYD, HYD
Hypo10	11.583	0.88	1231.8	4191.4	1102.3	HBA, HYD-AL, HYD-Ar, HYD, HYD

^a^ Root Mean Square Deviation; ^b^ squared correlation coefficient; ^c^ overall cost parameter of the PH4 pharmacophore; ^d^ cost difference between Null cost and total cost of this hypothesis; ^e^ lowest cost of 49 scrambled runs at a selected level of confidence of 98%. Fixed Cost = 15.2 with RMSD = 0, Null Cost = 5423.2 with RMSD = 24.53 and Configuration cost = 9.55. ^f^ HBA (hydrogen-bond Acceptor); HYD (Hydrophobic); HYD-AL (Hydrophobic Aliphatic); HYD-Ar (Hydrophobic Aromatic).

**Table 5:** $R_{1}$, $R_{2}$ and $R_{3}$ groups used in the design of the initial diversity library of TCL analogues.

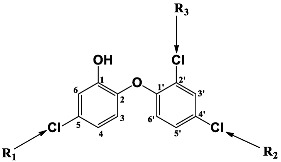					
R-Groups					
**1**	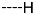	**2**	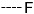	**3**	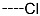
**4**	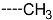	**5**	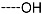	**6**	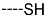
**7**	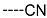	**8**	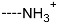	**9**	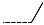
**10**	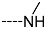	**11**	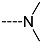	**12**	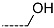
**13**	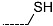	**14**	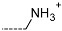	**15**	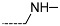
**16**	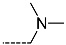	**17**	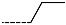	**18**	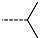
**19**	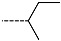	**20**	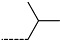	**21**	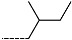
**22**	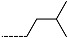	**23**	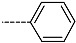	**24**	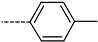
**25**	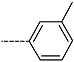	**26**	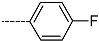	**27**	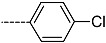
**28**	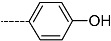	**29**	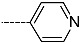	**30**	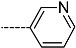
**31**	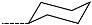	**32**	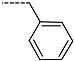	**33**	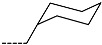
**34**	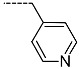	**35**	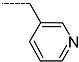	**36**	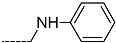
**37**	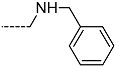	**38**	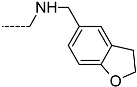	**39**	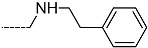
**40**	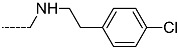	**41**	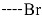	**42**	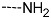
**43**	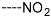	**44**	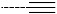	**45**	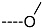
**46**	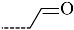	**47**	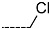	**48**	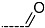
**49**	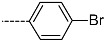	**50**	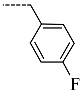	**51**	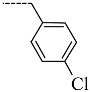
**52**	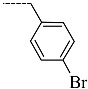	**53**	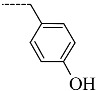	**54**	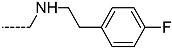
**55**	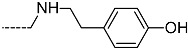	**56**	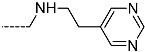	**57**	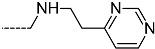
**58**	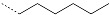	**59**	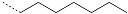	**60**	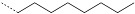

$R_{1}$-groups: fragments **1**–**60**; $R_{2}$-groups: fragments **1**–**23**; **41**–**48**. $R_{3}$-groups: fragments **1**–**11**; **41**–**47**. Dashed bonds indicate the attachment point of the fragment.

**Table 6:** Relative GFE calculated and its components for 20 best analogues of TCLs identified by in silico screening of the diversity combinatorial library. The numbering of the analogues concatenates the index of each substituent $R_{1}-R_{2}-R_{3}$ with the substituent numbers taken from Table 5.

No.	TCL Analogue	${∆∆H}_{MM}$ ^a^ ${[kcal\cdot mol}^{-1}]$	${∆∆G}_{sol}$ ^b^ ${[kcal\cdot mol}^{-1}]$	${∆∆TS}_{vib}$ ^c^ ${[kcal\cdot mol}^{-1}]$	${∆∆G}_{com}$ ^d^ $\left[{kcal\cdot mol}^{-1}\right]$	${pIC}_{50}^{pre}$ ^e^	${IC}_{50}^{pre}$^f^ [nM]
Ref.	**TCL1** **TCL-03-03-03**	0	0	0	0	7.14 *	73 *
1	**TCL-58-01-01**	−17.8	8.1	7.6	−17.3	7.87	13.4
2	**TCL-59-01-01**	−20.6	8.1	6.5	−19.0	7.97	10.8
3	**TCL-60-01-01**	−21.8	8.9	7.5	−20.4	8.04	9.1
8	**TCL-26-16-01**	−20.5	9.4	6.5	−17.6	7.89	12.8
47	**TCL-33-08-05**	−18.9	0.4	8.3	−26.7	8.39	4.1
51	**TCL-59-08-05**	−13.8	3.0	6.5	−17.3	7.87	13.4
52	**TCL-60-08-05**	−15.2	2.6	6.2	−18.8	7.96	11.1
85	**TCL-60-08-08**	−49.9	25.8	9.0	−33.0	8.73	1.9
91	**TCL-33-16-08**	−15.45	2.28	12.14	−25.3	8.31	4.9
92	**TCL-58-16-08**	−11.3	3.2	12.2	−20.3	8.04	9.2
94	**TCL-60-16-08**	−14.8	7.3	12.1	−19.6	8.00	10.1
112	**TCL-55-19-09**	0.8	−2.7	14.2	−16.1	7.81	15.5
118	**TCL-58-23-09**	−29.2	11.2	11.2	−29.2	8.52	3.1
119	**TCL-59-23-09**	−11.6	6.1	12.0	−17.4	7.88	13.2
135	**TCL-36-23-10**	−17.3	5.1	6.2	−18.4	7.93	11.6
154	**TCL-56-17-11**	−12.5	4.5	11.7	−19.6	8.00	10.1
155	**TCL-35-20-11**	−4.3	−1.7	11.6	−17.7	7.90	12.7
191	**TCL-32-08-45**	−17.1	3.5	4.3	−18.0	7.91	12.2
200	**TCL-56-16-46**	−14.0	4.2	7.6	−17.4	7.88	13.2
205	**TCL-56-23-46**	−21.7	6.1	7.1	−22.7	8.17	6.8

^a^${∆∆H}_{MM}$ is the relative enthalpic contribution to the GFE change ${∆∆G}_{com}$ of the *Pf*ENR-TCLx complex formation (for details, see the footnote in Table 2); ^b^ ${∆∆G}_{sol}$ is the relative solvation contribution to ${∆∆G}_{com}$; ^c^ ${∆∆TS}_{vib}$ is the relative entropic (vibrational) contribution to ${∆∆G}_{com}$; ^d^ ${∆∆G}_{com}$ is the relative GFE change of the *Pf*ENR-TCLx complex formation ${∆∆G}_{com}={∆∆H}_{MM}+{∆∆G}_{sol}-{∆∆TS}_{vib}$; ^e^ $p{IC}_{50}^{pre}$ is the logarithm of predicted inhibition potency towards *Pf*ENR calculated from ${∆∆G}_{com}$ using correlation Equation (2), Table 3; ^f^ ${IC}_{50}^{pre}$ is the predicted inhibition potency toward *Pf*ENR. * Experimental values of $p{IC}_{50}^{exp}$ and ${IC}_{50}^{exp}$ are given for the reference inhibitor **TCL1** instead of the predicted values.

**Table 7:** ADME-related properties of virtual TCL hits and other reference antimalarial agents computed by QikProp [39].

Analogues ^a^	# Star ^b^	$M_{W}$	S_mol_^d^	S_mol, hfo_^e^	V_m_^f^	RotB ^g^	HBD ^h^	HBA ^i^	logP_o/w_^j^	logS_wat_^k^	logKHSA ^l^	logB/B ^m^	BIP_caco_^n^	# Meta ^o^	${IC}_{50}^{pre}$*Pf*ENR [nM]	HOA ^q^	%HOA ^r^
58-01-01	1	270	592.4	259.8	1009.0	8	1.0	1.3	5.0	−5.2	0.8	−0.5	2933.7	2	13.3	3	100
59-01-01	1	284	622.3	291.2	1070.5	9	1.0	1.3	5.4	−5.7	0.9	−0.5	2936.4	2	10.8	3	100
60-01-01	1	298	654.7	322.1	1126.8	10	1.0	1.3	5.8	−6.1	1.0	−0.6	2933.7	2	9.1	3	100
26-16-01	0	337	652.3	181.2	1130.1	6	1.0	3.3	4.7	−5.0	0.8	0.2	759.3	3	12.9	3	100
29-16-01	0	320	635.4	181.3	1099.0	6	1.0	4.8	3.5	−3.9	0.4	−0.2	416.7	3	22.0	3	94
02-21-01	1	274	557.6	218.1	953.5	6	1.0	1.3	4.8	−5.2	0.7	−0.2	3200.1	2	20.2	3	100
05-21-01	0	272	562.4	218.0	961.2	7	2.0	2.0	4.7	−4.3	0.4	−0.9	965.7	3	18.5	3	100
55-23-01	1	411	787.1	107.3	1375.0	10	3.0	3.5	5.3	−6.0	1.0	−1.1	168.6	5	38.2	2	85
56-04-02	0	353	674.0	194.7	1166.8	8	2.0	5.8	3.2	−4.1	0.3	−0.7	181.1	6	73.3	3	86
40-23-02	3	448	809.8	106.3	1416.7	9	2.0	2.8	6.9	−7.6	1.5	−0.1	562.2	4	32.1	1	100
55-45-02	0	383	704.6	199.7	1228.9	10	3.0	4.3	4.0	−4.5	0.5	−0.8	183.5	6	35.5	3	91
34-14-03	0	341	629.0	88.1	1078.7	7	3.0	3.8	3.2	−3.9	0.3	−0.7	115.8	6	67.2	3	83
38-10-04	0	390	735.5	353.2	1292.5	8	3.0	4.5	4.3	−5.1	0.7	−0.5	333.5	8	67.2	3	100
57-20-04	0	392	776.1	391.3	1373.8	10	2.0	5.3	4.6	−5.5	0.8	−0.8	274.6	8	30.5	3	100
33-08-05	0	313	607.7	243.6	1060.9	7	3.5	3.0	3.2	−4.5	0.4	−1.5	265.6	4	4.1	3	89
58-08-05	0	301	624.3	259.8	1066.5	10	3.5	3.0	3.2	−4.3	0.2	−1.8	236.0	4	19.4	3	88
59-08-05	0	315	656.9	291.5	1126.4	11	3.5	3.0	3.6	−4.8	0.3	−1.9	244.3	4	13.3	3	90
60-08-05	0	329	686.6	322.1	1184.3	12	3.5	3.0	3.9	−5.1	0.5	−2.0	236.0	4	11.1	3	92
37-20-06	0	394	758.8	256.5	1334.3	10	2.8	3.3	5.6	−6.0	1.0	−0.2	560.6	6	51.6	3	96
49-16-07	0	423	708.2	180.3	1231.8	7	1.0	4.8	4.4	−6.2	0.7	−0.6	184.3	3	26.2	3	93
32-19-07	1	357	711.7	227.3	1248.1	8	1.0	2.8	5.6	−8.0	1.2	−1.2	729.9	3	41.7	1	100
58-08-08	0	302	628.7	259.8	1074.4	10	4	3.5	3.0	−4.2	0.2	−1.9	202.2	5	89.6	3	85
59-08-08	0	316	692.1	310.1	1164.7	11	4	3.5	3.6	−5.2	0.3	−2.0	223.4	5	40.2	3	89
60-08-08	0	330	690.1	322.1	1190.5	12	4	3.5	3.7	−5.0	0.4	−2.1	206.6	5	1.9	3	90
58-16-08	0	343	702.5	426.6	1230.8	11	2.5	4.3	3.7	−4.2	0.5	−1.0	169.6	6	9.2	3	88
59-16-08	0	357	756.6	479.9	1321.3	12	2.5	4.3	4.4	−5.0	0.7	−1.0	219.6	6	22.6	3	94
60-16-08	0	371	763.8	488.9	1347.0	13	2.5	4.3	4.5	−5.0	0.8	−1.2	173.2	6	10.0	3	93
40-13-09	0	428	777.9	268.0	1377.1	11	2.8	3.3	6.1	−6.5	1.1	−0.1	616.6	7	38.2	1	100
54-20-09	3	422	818.2	402.0	1465.9	11	2.0	2.8	6.7	−7.2	1.5	−0.3	606.3	6	16.9	1	100
32-17-10	0	339	669.4	453.1	1201.7	6	2.0	2.3	5.3	−6.4	1.1	−0.6	1787.7	6	57.1	1	100
07-22-10	0	310	650.9	307.0	1111.8	8	2.0	3.8	3.7	−6.3	0.5	−1.5	410.8	5	22.3	1	95
58-23-09	4	374	739.2	355.2	1326.4	10	1	1.3	7.2	−7.7	1.6	−0.5	3666.3	3	3.0	1	100
59-23-09	4	388	754.3	380.4	1382.8	11	1	1.3	7.6	−7.8	1.7	−0.6	3622.7	3	13.2	1	100
60-23-09	4	402	825.5	442.5	1470.6	12	1	1.3	8.1	−9.0	1.9	−0.8	3267.4	3	21.0	1	100
46-23-10	0	333	647.9	150.2	1118.0	7	2.0	4.3	3.7	−5.3	0.5	−1.4	407.7	4	50.3	3	96
58-23-10	1	375	754.9	338.5	1332.6	10	2	2.3	6.3	−7.3	1.3	−0.9	1715.4	4	45.0	1	100
59-23-10	2	389	781.0	368.7	1386.8	11	2	2.3	6.8	−7.7	1.4	−0.8	2514.6	4	32.9	1	100
60-23-10	3	403	824.9	407.6	1459.3	12	2	2.3	7.2	−8.3	1.5	−1.1	1825.5	4	22.9	1	100
40-09-11	0	391	782.8	365.6	1382.3	10	2.0	3.8	5.6	−6.1	1.1	−0.5	520.7	7	45.5	3	95
37-15-41	0	427	735.0	193.1	1275.9	9	3.0	4.3	4.1	−4.1	0.7	0.0	86.9	6	52.3	3	86
27-16-41	0	433	703.5	183.0	1218.1	6	1.0	3.3	5.6	−6.4	1.1	0.4	740.0	3	66.3	1	100
32-08-45	0	321	605.6	114.4	1055.1	7	2.5	3.0	4.0	−4.8	0.5	−0.9	879.5	4	12.2	3	100
25-07-46	1	343	668.1	143.9	1151.1	7	1.0	4.8	3.5	−6.6	0.5	−1.9	136.3	3	16.5	1	86
28-13-47	0	373	656.3	93.6	1135.4	7	2.8	2.5	5.1	−6.3	0.7	−0.6	994.2	5	13.0	1	100
Chloroquine	1	294	594.1	188.9	982.9	6	0.0	3.0	4.6	−5.3	0.4	−0.1	3718.1	0	-	3	100
Amodiaquine	1	334	603.2	131.7	1018.7	6	0.0	5.0	3.6	−4.4	0.0	−0.4	1689.1	0	-	3	100
Dapsone	1	236	431.6	0.0	687.9	2	0.0	7.0	−0.4	−0.5	−1.3	−0.9	289.1	0	-	3	69
Trimethoprim	0	272	500.2	223.9	835.9	5	0.0	6.5	0.6	−1.5	−0.9	−1.2	282.8	3	-	3	74
Mefloquine	2	362	533.1	0.0	925.1	2	0.0	4.0	4.1	−4.9	0.2	0.5	2903.1	0	-	3	100
Pamaquine	0	315	654.8	443.4	1148.1	9	1.0	4.8	4.0	−3.8	0.4	0.2	1475.2	5	-	3	100
Sulfametopyrazine	1	268	473.4	77.8	773.3	4	0.0	9.0	−1.0	0.2	−1.7	−1.3	195.8	1	-	2	62
Quinacrine	0	370	680.5	268.8	1163.6	7	0.0	3.5	5.6	−6.5	0.8	−0.1	4435.7	1	-	1	100
Tetracycline	5	422	604.5	173.1	1111.8	2	0.0	7.0	−3.4	1.1	−2.5	−2.6	6.8	5	-	1	22
Lumefantrine	5	497	819.1	160.7	1437.5	7	0.0	3.0	8.3	−10.0	1.7	0.2	4337.2	0	-	1	100
Bulaquine	0	369	560.2	360.2	1097.8	9	1.0	5.8	3.6	−3.0	0.1	−0.4	3099.7	7	-	3	100
Hydroxychloroquine	1	310	609.5	119.5	1006.5	6	0.0	5.0	3.4	−4.5	−0.1	−0.7	1023.7	0	-	3	100
Sulfadoxine	1	296	510.6	152.3	849.5	5	0.0	9.5	−0.8	−0.1	−1.7	−1.4	213.4	2	-	2	64
Halofantrine	5	470	785.4	160.2	1351.8	5	0.0	3.0	7.6	−9.9	1.5	0.2	2844.1	0	-	1	100
Proguanil	1	238	478.2	125.3	768.6	6	0.0	6.0	1.1	−1.5	−1.1	−0.7	834.6	0	-	3	86
Doxycycline	4	422	602.2	174.1	1104.2	2	0.0	17.2	−4.0	1.7	−2.9	−2.5	9.2	4	-	1	21
Artemether	1	312	531.1	506.1	970.2	2	0.0	5.7	2.7	−2.99	−0.20	0.20	5731.8	0	-	3	100
Dihydroartemisinine	1	284	477.4	395.7	864.6	1	1.0	5.7	1.8	−2.92	−0.10	−0.10	1664.9	0	-	3	95
Artemisinin	0	282	456.6	380.6	848.4	0	0.0	5.3	1.7	−2.10	−0.30	0.00	1886.0	1	-	3	96

^a^ designed TCL analogues (Table 6) and known antimalarials; ^b^ drug-likeness, number of property descriptors (24 of the full list of 49 QikProp descriptors, ver. 6.5, release 139) that fall outside the range of values for 95% of known drugs; ^c^ molar mass in [g·mol^−1^] (range for 95% of drugs: 130–725 g·mol^−1^) [39]; ^d^ total solvent-accessible molecular surface, in (Å^2^) (probe radius 1.4 Å) (range for 95% of drugs: 300–1000 Å^2^); ^e^ hydrophobic portion of the solvent-accessible molecular surface, in (Å^2^) (range for 95% of drugs: 0–750 Å^2^); ^f^ total volume of the molecule enclosed by solvent-accessible molecular surface, in (Å^3^) (range for 95% of drugs: 500–2000 Å^3^); ^g^ number of rotatable non-trivial (not CX3), non-hindered (not alkene, amide, small ring) bonds (range for 95% of drugs: 0–15); ^h^ estimated number of hydrogen bonds that would be donated by the solute to water molecules in an aqueous solution. Values are averages taken over a number of configurations, so they can assume non-integer values (range for 95% of drugs: 0.0–6.0); ^i^ estimated number of hydrogen bonds that would be accepted by the solute from water molecules in an aqueous solution (range for 95% of drugs: 2.0–20.0); ^j^ logarithm of the partition coefficient between the n-octanol and water phases (range for 95% of drugs: −2 to 6.5); ^k^ logarithm of predicted aqueous solubility, logS. S in (mol·dm^–3^) is the concentration of the solute in a saturated solution that is in equilibrium with the crystalline solid (range for 95% of drugs: −6.0 to 0.5); ^l^ logarithm of the predicted binding constant to human serum albumin (range for 95% of drugs: −1.5 to 1.5); ^m^ logarithm of the predicted brain/blood partition coefficient (range for 95% of drugs: −3.0 to 1.2); ^n^ predicted apparent Caco-2 cell membrane permeability on the Boehringer–Ingelheim scale in [nm·s^−1^] (range for 95% of drugs: <25 poor, >500 nm·s^−1^ great); ^o^ number of likely metabolic reactions (range for 95% of drugs: 1–8); ^p^ predicted inhibition constants ${IC}_{50}^{pre}$ of designed TCLs vs. *Pf*ENR.; ^q^ human oral absorption (1 = low, 2 = medium, 3 = high); ^r^ percentage of human oral absorption in the gastrointestinal tract (<25% = poor, >80% = high); * star in any column indicates that the property descriptor value of the compound falls outside the range of values for 95% of known drugs.

**Table 8:** Ensemble averages of the total and potential energies of complexes *Pf*ENR-TCLx for **TCL11** and selected virtual hits.

Inhibitor	Structure	$\langle E_{tot}\rangle$ ^a^ $\left[kcal\cdot {mol}^{-1}\right]$	$\langle E_{pot}\rangle$ ^b^ $\left[kcal\cdot {mol}^{-1}\right]$	${IC}_{50}^{pre}$^c^ [nM]
TCL11 *	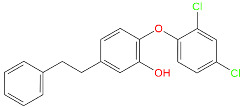	−113,962.1	−142,462.7	76
**TCL-33-08-05**	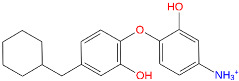	−114,523.3	−143,133.8	4.1
**TCL-58-01-01**	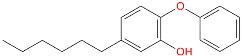	−116,055.6	−145,058.2	13.3
**TCL-58-16-08**	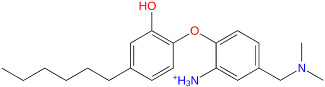	−114,528.9	−143,152.9	9.2
**TCL-59-01-01**	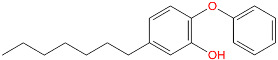	−114,445.5	−143,063.9	10.8
**TCL-60-01-01**	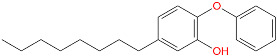	−114,415.0	−143,027.9	9.1

^a^ Ensemble average of the total energy $E_{tot}$ of the system (sum of potential energy $E_{pot}$ and kinetic energy $E_{kin}$); ^b^ Ensemble average of the potential energy $E_{pot}$; ^c^ ${IC}_{50}$ was predicted by QSAR model (Table 3, Equation (2)) for the designed analogues. Experimental ${IC}_{50}^{exp}$ for the **TCL11**. * **TCL11** is the co-crystallized ligand in the (PDB entry 1OOS) [21].

**Table 9:** Molecular dynamics system setup.

Inhibitors of *Pf*ENR	Number of Water Molecules	Number of Counterions	Number of Atoms of the System
**TCL11**	13,747	5 Cl^−^	45,561
**TCL-33-08-05**	13,806	6 Cl^−^	46,746
**TCL-58-01-01**	14,028	5 Cl^−^	47,406
**TCL-58-16-08**	13,810	6 Cl^−^	46,767
**TCL-59-01-01**	13,812	5 Cl^−^	46,761
**TCL-60-01-01**	13,807	5 Cl^−^	46,749

## Data Availability

The data presented in this study are available in this article and Supplementary Material.

## Supplementary Materials

The following supporting information can be downloaded at: https://www.mdpi.com/article/10.3390/ijms24086916/s1.
