# Supplementary material for: Structure-Based Design and Pharmacophore-Based Virtual Screening of Combinatorial Library of Triclosan Analogues Active against Enoyl-Acyl Carrier Protein Reductase of Plasmodium falciparum with Favourable ADME Profiles
Source: Int J Mol Sci. 2023 Apr 7;24(8):6916. doi: 10.3390/ijms24086916 (PMC10139228; doi:10.3390/ijms24086916)
Supplement: Supplementary file 1 [file ijms-24-06916-s001.zip › ijms-2307707-supplementary.pdf]

International Journal of *Molecular Sciences*

MDPI

Article

Structure-Based Design and Pharmacophore-Based Virtual Screening of Combinatorial Library of Triclosan Analogues Active against Enoyl-Acyl Carrier Protein Reductase of *Plasmodium falciparum* with Favourable ADME Profiles

Cecile Bieri [1], Akori Esmel [1], Melalie Keita [1], Luc Calvin Owono Owono [2,3], Brice Dali [1], Eugene Megnassan [1,3,4,5,6], Stanislav Miertus [3,7] and Vladimir Frecer [8,*]

1 Laboratoire de Physique Fondamentale et Appliquée (LPFA), University of Abobo Adjamé (Now Nangui Abrogoua), Abidjan 02, Côte d'Ivoire
2 Department of Physics, Ecole Normale Supérieure, University of Yaoundé I, P.O. Box 47, Yaoundé 1, Cameroon
3 International Centre for Applied Research and Sustainable Technology, SK-84104 Bratislava, Slovakia
4 International Centre for Theoretical Physics, Strada Costiera 11, I-34151 Trieste, Italy
5 Laboratoire de Cristallographie—Physique Moléculaire, Université De Cocody, Abidjan 22, Côte d'Ivoire
6 Laboratoire de Chimie Organique Structurale et Théorique, Université de Cocody, Abidjan 22, Côte d'Ivoire
7 Department of Biotechnologies, Faculty of Natural Sciences, University of SS. Cyril and Methodius, SK-91701 Trnava, Slovakia
8 Department of Physical Chemistry of Drugs, Faculty of Pharmacy, Comenius University Bratislava, SK-83232 Bratislava, Slovakia
* Correspondence: frecer@fpharm.uniba.sk

Table S1. Relative GFE calculated and its components for 219 analogues of TCLs identified by *in silico* screening of the diversity combinatorial library. The numbering of the analogues concatenates the index of each substituent $R_1 - R_2 - R_3$ with the substituent numbers taken from Tab. 5.

No.	TCL Analogue	$\Delta\Delta H_{MM}$ [a] [kcal · mol⁻¹]	$\Delta\Delta G_{sol}$ [b] [kcal · mol⁻¹]	$\Delta\Delta TS_{vib}$ [c] [kcal · mol⁻¹]	$\Delta\Delta G_{com}$ [d] [kcal · mol⁻¹]	pIC_{50}^{pre} [e]	IC_{50}^{pre} [f] [nM]
Ref.	TCL1/03-03-03	0	0	0	0	7.14 *	73 *
1	58-01-01	-17.8	8.1	7.6	-17.3	7.87	13.4
2	59-01-01	-20.6	8.1	6.5	-19.0	7.97	10.8
3	60-01-01	-21.8	8.9	7.5	-20.4	8.04	9.1
4	37-12-01	3.3	2.2	7.1	-1.6	7.02	95.4
5	47-13-01	10.8	-2.8	4.3	3.7	6.73	185.2
6	47-14-01	23.4	-5.9	7.4	10.1	6.38	412.9
7	25-16-01	7.1	-2.1	8.5	-3.4	7.12	76.1
8	26-16-01	-20.5	9.4	6.5	-17.6	7.89	12.8
9	29-16-01	-15.4	9.6	7.5	-13.3	7.66	22.1
10	40-16-01	10.3	-5.8	10.3	-5.8	7.25	56.4
11	32-20-01	1.2	3.2	6.4	-2.0	7.04	90.7
12	02-21-01	-12.4	1.0	1.0	-14.0	7.70	20.2
13	05-21-01	-12.9	7.1	8.9	-14.7	7.73	18.5
14	44-21-01	11.7	-0.6	8.4	2.8	6.78	165.5
15	35-23-01	-0.9	3.3	4.2	-1.8	7.03	93.1
16	44-23-01	9.2	1.3	5.6	4.8	6.67	212.6

17	55-23-01	-1.0	-1.6	6.3	-8.9	7.42	38. 2
18	22-41-01	10.1	-1.8	6.1	2.2	6.81	153.5
19	56-04-02	2.9	-2.9	3.7	-3.7	7.13	73.3
20	40-08-02	13.9	-8.8	7.0	-1.9	7.04	91.8
21	37-12-02	0.1	0.9	4.9	-3.8	7.14	72.4
22	45-12-02	9.5	0.5	6.1	4.0	6.72	192.3
23	38-13-02	1.0	1.3	6.0	-3.7	7.13	73.3
24	56-14-02	-3.3	-1.5	2.8	-7.6	7.35	45.1
25	55-16-02	2.2	-1.8	9.0	-8.6	7.40	39.7
26	50-17-02	0.4	2.3	5.5	-2.8	7.09	82.1
27	07-19-02	10.7	1.1	5.5	6.3	6.59	256.5
28	54-21-02	1.2	-2.4	10.6	-11.8	7.58	26.6
29	40-23-02	-2.4	-0.6	7.3	-10.3	7.49	32.1
30	55-45-02	0.2	-3.3	6.3	-9.5	7.45	35.5
31	39-48-02	1.0	-0.3	5.2	-4.5	7.18	66.3
32	37-07-03	0.0	1.1	3.3	-2.2	7.05	88.5
33	34-14-03	-1.3	1.1	4.2	-4.4	7.17	67.2
34	05-20-03	11.1	1.5	5.6	7.1	6.55	283.6
35	41-22-03	9.8	-0.7	6.6	2.5	6.80	159.4
36	21-42-03	7.2	-0.6	5.2	1.4	6.86	138.9
37	27-46-03	0.2	-0.6	0.4	-0.8	6.98	105.4
38	55-04-04	0.1	-2.4	5.5	-7.8	7.36	43.9
39	53-09-04	1.0	2.5	4.9	-1.4	7.01	97.8
40	38-10-04	2.7	0.5	7.5	-4.4	7.17	67.2
41	04-12-04	9.1	-1.6	4.8	2.8	6.78	165.5
42	52-12-04	-1.4	2.1	1.3	-0.6	6.97	108.1
43	46-17-04	9.2	0.2	4.8	4.6	6.68	207.3
44	57-20-04	-1.8	2.1	11.1	-10.7	7.52	30.5
45	37-42-04	3.5	0.7	3.8	0.5	6.91	124.1
46	55-01-05	3.1	-3.5	3.8	-4.2	7.16	68.9
47	33-08-05	-18.9	0.4	8.3	-26.7	8.39	4.1
48	39-08-05	-1.1	-3.7	4.0	-8.8	7.41	38.7
49	56-08-05	0.2	-4.5	5.5	-9.8	7.47	34.2
50	58-08-05	-11.3	3.1	6.0	-14.3	7.71	19.4
51	59-08-05	-13.8	3.0	6.5	-17.3	7.87	13.4
52	60-08-05	-15.2	2.6	6.2	-18.8	7.96	11.1
53	40-13-05	5.4	-4.3	1.8	-0.7	6.97	106.7
54	16-23-05	1.2	2.3	4.9	-1.4	7.01	97.8
55	09-48-05	8.1	0.5	4.8	3.7	6.73	185.2
56	21-02-06	7.2	-0.8	5.9	0.5	6.91	124.1
57	21-11-06	6.1	0.4	10.7	-4.3	7.17	68.1
58	16-14-06	21.7	-3.7	5.9	12.1	6.28	530.4
59	12-15-06	6.8	-0.2	2.9	3.7	6.73	185.2
60	47-15-06	6.7	0.0	5.3	1.4	6.86	138.9

61	37-20-06	-4.3	3.7	5.9	-6.5	7.29	51.6
62	08-21-06	19.4	-4.8	7.5	7.1	6.55	283.6
63	44-21-06	9.2	-0.6	5.6	3.1	6.76	171.8
64	07-23-06	10.0	2.4	2.2	10.3	6.37	423.4
65	16-23-06	8.5	-0.1	4.2	4.2	6.71	197.2
66	12-47-06	8.9	-1.2	1.8	5.9	6.61	244.1
67	39-47-06	2.3	0.3	0.7	1.9	6.83	147.8
68	38-04-07	2.4	-0.5	3.7	-1.8	7.03	93.1
69	56-08-07	0.6	-4.2	3.9	-7.5	7.34	45.5
70	57-14-07	-2.5	-4.8	1.0	-8.2	7.38	41.7
71	04-15-07	12.1	-2.8	4.9	4.4	6.69	202.2
72	49-16-07	-19.1	9.7	2.5	-11.9	7.58	26.2
73	01-17-07	15.5	-3.0	5.3	7.3	6.54	290.8
74	56-18-07	4.3	-0.5	7.3	-3.6	7.13	74.3
75	32-19-07	-2.9	2.4	7.6	-8.2	7.38	41.7
76	13-21-07	11.6	-0.5	3.2	7.9	6.50	313.4
77	06-23-07	9.0	0.0	3.6	5.3	6.65	226.3
78	56-41-07	3.6	-1.8	1.4	0.4	6.91	122.5
79	31-42-07	9.7	0.0	8.3	1.4	6.86	138.9
80	56-07-08	16.9	-9.7	5.8	1.4	6.86	138.9
81	36-08-08	-1.2	4.4	4.8	-1.6	7.02	95.4
82	57-08-08	-2.4	1.3	7.4	-8.4	7.39	40.7
83	58-08-08	-0.7	8.8	10.2	-2.1	7.05	89.6
84	59-08-08	-6.2	8.1	10.4	-8.5	7.40	40.2
85	60-08-08	-49.9	25.8	9.0	-33.0	8.73	1.9
86	57-12-08	12.5	-8.8	6.6	-2.9	7.09	81.1
87	19-13-08	22.5	-6.1	6.5	10.0	6.39	407.8
88	37-15-08	17.2	-6.8	4.6	5.7	6.62	237.9
89	46-15-08	19.6	-7.4	7.3	4.9	6.67	215.3
90	02-16-08	23.8	-5.4	5.0	13.4	6.20	624.3
91	33-16-08	-15.45	2.28	12.14	-25.3	8.31	4.9
92	58-16-08	-11.3	3.2	12.2	-20.3	8.04	9.2
93	59-16-08	-6.4	3.0	9.8	-13.1	7.65	22.6
94	60-16-08	-14.8	7.3	12.1	-19.6	8.00	10.1
95	30-20-08	9.4	-3.4	5.6	0.5	6.91	124.1
96	55-20-08	19.7	-10.2	10.7	-1.2	7.00	100.3
97	12-21-08	28.8	-6.3	8.4	14.2	6.16	690.1
98	29-21-08	15.9	-5.1	6.8	4.0	6.72	192.3
99	30-21-08	16.7	-8.2	5.3	3.2	6.76	174.1
100	21-22-08	19.8	-3.6	14.5	1.6	6.85	142.4
101	03-23-08	17.2	-5.0	1.9	10.3	6.37	423.4
102	09-23-08	18.7	-3.3	3.4	12.0	6.28	523.8
103	16-23-08	18.1	-6.5	9.2	2.4	6.80	157.4
104	17-23-08	16.8	-4.6	6.7	5.5	6.63	232.1

105	46-23-08	17.9	-3.7	3.8	10.3	6.37	423.4
106	55-47-08	16.2	-12.8	6.5	-3.1	7.10	79.1
107	32-02-09	3.8	0.1	2.7	1.2	6.87	135.4
108	29-04-09	3.9	1.6	4.2	1.4	6.86	138.9
109	40-06-09	7.2	-6.2	3.6	-2.6	7.08	84.2
110	05-10-09	6.1	-0.1	8.2	-2.2	7.05	88.5
111	40-13-09	4.4	-4.3	9.0	-8.9	7.42	38.2
112	55-19-09	0.8	-2.7	14.2	-16.1	7.81	15.5
113	24-20-09	0.5	1.9	5.5	-3.1	7.10	79.1
114	54-20-09	-1.5	-0.9	13.0	-15.4	7.77	16.9
115	42-22-09	16.4	-2.8	9.2	4.4	6.69	202.2
116	25-23-09	-5.8	1.6	4.0	-8.3	7.39	41.2
117	36-23-09	-6.5	2.2	8.7	-13.0	7.64	22.9
118	58-23-09	-29.2	11.2	11.2	-29.2	8.52	3.1
119	59-23-09	-11.6	6.1	12.0	-17.4	7.88	13.2
120	60-23-09	-3.2	0.2	10.8	-13.7	7.68	21.1
121	26-01-10	5.5	-0.2	4.1	1.1	6.87	133.7
122	53-02-10	-4.0	-5.2	3.0	-12.3	7.60	25.1
123	26-10-10	0.4	0.8	6.0	-4.8	7.19	63.9
124	35-10-10	2.2	8.5	5.4	5.3	6.65	226.3
125	40-14-10	17.5	-10.5	6.8	0.2	6.92	119.5
126	31-17-10	5.8	0.5	12.1	-5.7	7.24	57.1
127	36-18-10	-6.0	0.8	8.6	-13.9	7.69	20.4
128	02-20-10	9.7	-1.2	7.6	0.9	6.88	130.4
129	18-20-10	9.3	-0.9	10.9	-2.5	7.07	85.2
130	28-21-10	1.3	0.6	9.1	-7.3	7.33	46.7
131	07-22-10	-6.9	1.0	7.3	-13.2	7.65	22.3
132	13-22-10	17.5	-2.2	8.1	7.2	6.54	287.1
133	07-23-10	6.3	0.0	5.6	0.7	6.90	127.2
134	19-23-10	2.1	0.4	8.6	-6.1	7.27	54.3
135	36-23-10	-17.3	5.1	6.2	-18.4	7.93	11.6
136	44-23-10	4.8	0.2	4.3	0.6	6.90	125.6
137	46-23-10	-10.9	7.8	3.6	-6.7	7.30	50.3
138	58-23-10	-0.5	2.9	10.1	-7.6	7.35	45.1
139	59-23-10	-3.1	3.7	10.8	-10.1	7.48	32.9
140	60-23-10	-1.2	1.5	13.2	-13.0	7.64	22.9
141	47-44-10	7.0	-1.8	5.1	0.1	6.93	118.1
142	53-05-11	0.0	3.8	6.6	-2.8	7.09	82.1
143	39-06-11	2.0	0.3	5.0	-2.7	7.08	83.1
144	37-09-11	3.6	-0.2	6.4	-3.0	7.10	80.1
145	40-09-11	3.5	1.6	12.6	-7.5	7.34	45.5
146	35-11-11	3.2	5.1	7.6	0.7	6.90	127.2
147	55-12-11	2.1	-2.3	8.6	-8.8	7.41	38.7
148	14-13-11	16.7	-3.3	5.7	7.6	6.52	301.9

149	21-13-11	9.3	1.1	7.4	3.0	6.77	169.7
150	29-13-11	4.8	2.1	2.8	4.1	6.71	194.7
151	28-16-11	-0.2	3.5	8.3	-5.0	7.21	62.3
152	28-17-11	-3.6	2.2	8.4	-9.8	7.47	34.1
153	44-17-11	5.9	1.0	6.6	0.3	6.92	121.1
154	56-17-11	-12.5	4.5	11.7	-19.6	8.00	10.1
155	35-20-11	-4.3	-1.7	11.6	-17.7	7.90	12.7
156	46-20-11	7.4	0.9	9.3	-1.0	6.99	102.8
157	49-21-11	5.6	0.0	10.0	-4.4	7.17	67.2
158	25-23-11	-8.2	3.4	4.5	-9.2	7.43	36.8
159	57-41-11	2.6	0.9	6.1	-2.6	7.08	84.1
160	49-45-11	-0.2	0.4	4.9	-4.7	7.19	64.7
161	48-05-41	9.8	0.2	4.3	5.7	6.62	238.1
162	32-15-41	-2.8	2.8	3.0	-2.9	7.09	81.1
163	37-15-41	-0.8	0.8	6.4	-6.4	7.28	52.3
164	27-16-41	-1.2	0.8	4.1	-4.5	7.18	66.3
165	25-19-41	7.1	-2.6	5.6	-1.1	6.99	101.5
166	28-21-41	-2.8	4.0	5.8	-4.5	7.18	66.3
167	17-08-42	-4.5	3.6	7.3	-8.1	7.37	42.2
168	33-15-42	1.3	2.6	11.8	-7.8	7.36	43.9
169	19-17-42	8.8	-1.2	10.6	-2.9	7.09	81.1
170	27-20-42	1.0	4.1	5.3	-0.2	6.94	113.6
171	07-23-42	9.6	2.9	4.5	8.1	6.49	321.4
172	09-23-42	7.5	2.6	7.8	2.3	6.81	155.4
173	44-23-42	9.3	3.4	6.2	6.5	6.58	263.1
174	57-47-42	3.3	-1.2	5.2	-3.1	7.10	79.1
175	53-01-44	1.4	1.5	4.9	-2.0	7.04	90.7
176	52-06-44	1.0	0.9	0.6	1.3	6.86	137.1
177	26-08-44	0.5	-1.1	1.8	-2.4	7.06	86.3
178	52-09-44	-1.1	0.6	2.2	-2.7	7.08	83.1
179	31-13-44	7.8	-0.7	7.0	0.1	6.93	118.1
180	05-15-44	12.0	0.4	6.5	6.0	6.61	247.1
181	13-16-44	6.3	4.6	5.8	5.1	6.66	220.7
182	45-17-44	9.3	0.2	7.6	1.9	6.83	147.8
183	35-21-44	6.4	-4.7	6.6	-5.0	7.21	62.3
184	17-23-44	3.3	-1.2	4.6	-2.5	7.07	85.2
185	41-23-44	5.5	-0.1	4.3	1.1	6.87	133.7
186	45-23-44	4.5	1.2	6.3	-0.5	6.96	109.5
187	37-41-44	0.6	0.4	4.1	-3.2	7.11	78.1
188	57-44-44	2.0	-1.7	3.9	-3.7	7.13	73.3
189	26-47-44	0.7	0.0	1.1	-0.4	6.96	110.8
190	21-06-45	6.5	-0.4	4.9	1.2	6.87	135.4
191	32-08-45	-17.1	3.5	4.3	-18.0	7.91	12.2
192	55-09-45	3.8	-3.8	7.9	-7.9	7.36	43.3

193	05-13-45	15.2	0.1	-1.9	17.1	6.00	992.3
194	40-23-45	-5.0	2.6	9.1	-11.5	7.56	27.6
195	53-23-45	-10.6	4.3	3.8	-10.0	7.48	33.3
196	13-45-45	8.4	-0.3	6.0	2.1	6.82	151.6
197	25-07-46	-16.1	4.2	3.7	-15.6	7.78	16.5
198	57-10-46	-1.4	1.6	7.0	-6.8	7.30	49.7
199	24-14-46	-7.5	-1.9	5.5	-14.9	7.74	18.1
200	56-16-46	-14.0	4.2	7.6	-17.4	7.88	13.2
201	06-23-46	-19.4	14.9	4.7	-9.2	7.43	36.8
202	07-23-46	2.6	0.4	3.8	-0.8	6.98	105.4
203	17-23-46	-3.2	1.6	5.1	-6.7	7.30	50.3
204	45-23-46	-15.5	4.9	1.9	-12.5	7.61	24.3
205	56-23-46	-21.7	6.1	7.1	-22.7	8.17	6.8
206	13-45-46	4.7	-0.4	4.2	0.1	6.93	118.1
207	25-45-46	-17.5	5.4	4.1	-16.2	7.81	15.3
208	18-46-46	7.5	-1.0	5.7	0.8	6.89	128.8
209	13-47-46	5.1	-0.7	2.8	1.6	6.85	142.4
210	55-07-47	-2.4	-2.3	2.7	-7.3	7.33	46.7
211	46-08-47	3.9	-1.6	2.4	-0.1	6.94	115.1
212	20-12-47	4.9	0.7	4.3	1.4	6.86	138.9
213	28-13-47	-25.3	8.3	0.4	-17.5	7.89	13.1
214	45-15-47	-16.4	5.6	3.8	-14.6	7.73	18.7
215	55-15-47	-2.4	-0.1	6.1	-8.6	7.40	39.7
216	02-16-47	9.8	0.4	3.1	7.1	6.55	283.6
217	20-20-47	2.8	-0.2	10.8	-8.2	7.38	41.7
218	25-42-47	2.8	-0.7	2.7	-0.7	6.97	106.7
219	22-48-47	4.0	-0.4	3.5	0.1	6.93	118.1

[a] $\Delta\Delta H_{MM}$ is the relative enthalpic contribution to the GFE change $\Delta\Delta G_{com}$ of the *Pf*ENR - TCLx complex formation (for details, see the footnote in Tab. 2);

[b] $\Delta\Delta G_{sol}$ is the relative solvation contribution to $\Delta\Delta G_{com}$;

[c] $\Delta\Delta TS_{vib}$ is the relative entropic (vibrational) contribution to $\Delta\Delta G_{com}$;

[d] $\Delta\Delta G_{com}$ is the relative GFE change of the *Pf*ENR - TCLx complex formation $\Delta\Delta G_{com} = \Delta\Delta H_{MM} + \Delta\Delta G_{sol} - \Delta\Delta TS_{vib}$; details of the rGFE calculation can be found in ref.: Frecer, V., Miertus, S., Tossi, A., Romeo, D. Rational design of inhibitors for drug-resistant HIV-1 aspartic protease mutants. *Drug Design and Discovery* **1998**, 15, 211-231.

[e] pIC_{50}^{pre} is the logarithm of predicted inhibition potency towards *Pf*ENR calculated from $\Delta\Delta G_{com}$ using correlation eq. 2, Tab. 3;

[f] IC_{50}^{pre} is the predicted inhibition potency toward *Pf*ENR;

[*] Experimental values of pIC_{50}^{exp} and IC_{50}^{exp} are given for the reference inhibitor TCL1 instead of the predicted values.
